# SHEA/IDSA/APIC Practice Recommendation: Strategies to prevent methicillin-resistant *Staphylococcus aureus* transmission and infection in acute-care hospitals: 2022 Update

**DOI:** 10.1017/ice.2023.102

**Published:** 2023-07

**Authors:** Kyle J. Popovich, Kathy Aureden, D. Cal Ham, Anthony D. Harris, Amanda J. Hessels, Susan S. Huang, Lisa L. Maragakis, Aaron M. Milstone, Julia Moody, Deborah Yokoe, David P. Calfee

**Affiliations:** 1 Department of Internal Medicine, RUSH Medical College, Chicago, Illinois; 2 Infection Prevention, Advocate Aurora Health, Downers Grove, Illinois; 3 Centers for Disease Control and Prevention, Atlanta, Georgia; 4 Health Care Outcomes Research, University of Maryland School of Medicine, Baltimore, Maryland; 5 Columbia School of Nursing, New York, New York; 6 Hackensack Meridian Health, Edison, New Jersey; 7 Division of Infectious Diseases, University of California Irvine School of Medicine, Irvine, California; 8 Johns Hopkins University School of Medicine, The Johns Hopkins Hospital, Baltimore, Maryland; 9 Division of Pediatric Infectious Diseases, Johns Hopkins University School of Medicine, Baltimore, Maryland; 10 Infection Prevention, HCA Healthcare, Nashville, Tennessee; 11 Department of Medicine, University of California San Francisco School of Medicine, San Francisco, California; 12 Transplant Infectious Diseases, UCSF Medical Center, San Francisco, California; 13 Department of Medicine, Weill Cornell Medicine, New York, New York; 14 Department of Population Health Sciences, Weill Cornell Medicine, New York, New York

## Abstract

Previously published guidelines have provided comprehensive recommendations for detecting and preventing healthcare-associated infections (HAIs). The intent of this document is to highlight practical recommendations in a concise format designed to assist acute-care hospitals in implementing and prioritizing efforts to prevent methicillin-resistant *Staphylococcus aureus* (MRSA) transmission and infection. This document updates the “Strategies to Prevent Methicillin-Resistant *Staphylococcus aureus* Transmission and Infection in Acute Care Hospitals” published in 2014.^1^ This expert guidance document is sponsored by the Society for Healthcare Epidemiology of America (SHEA). It is the product of a collaborative effort led by SHEA, the Infectious Diseases Society of America (IDSA), the Association for Professionals in Infection Control and Epidemiology (APIC), the American Hospital Association (AHA), and The Joint Commission, with major contributions from representatives of a number of organizations and societies with content expertise.

## Purpose

Previously published guidelines have provided comprehensive recommendations for detecting and preventing healthcare-associated infections (HAIs). The intent of this document is to highlight practical recommendations in a concise format designed to assist acute-care hospitals in implementing and prioritizing efforts to prevent methicillin-resistant *Staphylococcus aureus* (MRSA) transmission and infection. This document updates the “Strategies to Prevent Methicillin-Resistant *Staphylococcus aureus* Transmission and Infection in Acute Care Hospitals” published in 2014.^1^ This expert guidance document is sponsored by the Society for Healthcare Epidemiology of America (SHEA). It is the product of a collaborative effort led by SHEA, the Infectious Diseases Society of America (IDSA), the Association for Professionals in Infection Control and Epidemiology (APIC), the American Hospital Association (AHA), and The Joint Commission, with major contributions from representatives of a number of organizations and societies with content expertise.

## Summary of major changes

This section lists major changes from the “Strategies to Prevent Methicillin-Resistant *Staphylococcus aureus* Transmission and Infection in Acute Care Hospitals” published in 2014,^1^ including recommendations that have been added, removed, or altered. Recommendations are categorized as essential practices that should be adopted by all acute-care hospitals (in 2014 these were “basic practices,” renamed to highlight their importance as foundational for HAI prevention programs) or additional approaches that can be considered for use in locations and/or populations within hospitals when transmission or infection from MRSA is not controlled after implementation of essential practices (in 2014 these were “special approaches”). See Table 1 for a complete summary of the recommendations contained in this document.

**Table 1.** Summary of Recommendations to Prevent MRSA Infection and Transmission

Essential practices	
**1**	Implement a MRSA monitoring program. (Quality of evidence: LOW)
**2**	Conduct a MRSA risk assessment. (Quality of evidence: LOW)
**3**	Promote compliance with the CDC or WHO hand hygiene recommendations. (Quality of evidence: MODERATE)
**4**	Use contact precautions for MRSA-colonized and MRSA-infected patients. A facility that chooses or has already chosen to modify the use of contact precautions for some or all of these patients should conduct a MRSA-specific risk assessment to evaluate the facility for transmission risks and to assess the effectiveness of other MRSA risk mitigation strategies (eg, hand hygiene, cleaning and disinfection of the environment, single occupancy patient rooms), and establish a process for ongoing monitoring, oversight, and risk assessment. (Quality of evidence: MODERATE)
**5**	Ensure cleaning and disinfection of equipment and the environment. (Quality of evidence: MODERATE)
**6**	Implement a laboratory-based alert system that notifies HCP of new MRSA-colonized or MRSA-infected patients in a timely manner. (Quality of evidence: LOW)
**7**	Implement an alert system that identifies readmitted or transferred MRSA-colonized or MRSA-infected patients. (Quality of evidence: LOW)
**8**	Provide MRSA data and outcome measures to key stakeholders, including senior leadership, physicians, nursing staff, and others. (Quality of evidence: LOW)
**9**	Educate healthcare personnel about MRSA. (Quality of evidence: LOW)
**10**	Educate patients and families about MRSA. (Quality of evidence: LOW)
**11**	Implement an antimicrobial stewardship program. (Quality of evidence: LOW)

Additional approaches	
*Active surveillance testing (AST)*	
**1**	Implement a MRSA AST program for select patient populations as part of a multifaceted strategy to control and prevent MRSA. (Quality of evidence: MODERATE). Note: Specific populations may have different evidence ratings.
**2**	Active surveillance for MRSA in conjunction with decolonization can be performed in targeted populations prior to surgery to prevent post-surgical MRSA infection. (Quality of evidence: MODERATE)
**3**	Active surveillance with contact precautions is inferior to universal decolonization for reduction of MRSA clinical isolates in adult ICUs. (Quality of evidence: HIGH)
**4**	Hospital-wide active surveillance for MRSA can be used in conjunction with contact precautions to reduce the incidence of MRSA infection. (Quality of evidence: MODERATE)
**5**	Active surveillance can be performed in the setting of a MRSA outbreak or evidence of ongoing transmission of MRSA within a unit as part of a multifaceted strategy to halt transmission. (Quality of evidence: MODERATE)
*Screen healthcare personnel (HCP) for MRSA infection or colonization*	
**1**	Screen HCP for MRSA infection or colonization if they are epidemiologically linked to a cluster of MRSA infections. (Quality of evidence: LOW)
*MRSA decolonization therapy*	
**1**	Use universal decolonization (daily CHG bathing plus 5 days of nasal decolonization) for all patients in adult ICUs to reduce endemic MRSA clinical cultures. (Quality of evidence: HIGH)
**2**	Perform preoperative nares screening with targeted use of CHG and nasal decolonization in MRSA carriers to reduce MRSA SSI, in surgical procedures involving implantation of hardware. (Quality of evidence: MODERATE)
**3**	Screen for MRSA and provide targeted decolonization with CHG bathing and nasal decolonization to MRSA carriers in surgical units to reduce postoperative MRSA inpatient infections. (Quality of evidence: MODERATE)
**4**	Provide CHG bathing plus nasal decolonization to known MRSA carriers outside the ICU with medical devices, specifically central lines, midline catheters, and lumbar drains, to reduce MRSA clinical cultures. (Quality of evidence: MODERATE)
**5**	Consider postdischarge decolonization of MRSA carriers to reduce postdischarge MRSA infection and readmission. (Quality of evidence: HIGH)
**6**	Neonatal ICUs should consider targeted or universal decolonization during times of above-average MRSA infection rates or targeted decolonization for patients at high risk of MRSA infection (eg, low birthweight, indwelling devices, or prior to high-risk surgeries). (Quality of evidence: MODERATE)
**7**	Burn units should consider targeted or universal decolonization during times of above average MRSA infection rates. (Quality of evidence: MODERATE)
**8**	Consider targeted or universal decolonization of hemodialysis patients. (Quality of evidence: MODERATE)
**9**	Decolonization should be strongly considered as part of a multimodal approach to control MRSA outbreaks. (Quality of evidence: MODERATE)
*Universal use of gowns and gloves*	
**1**	Use gowns and gloves when providing care to or entering the room of all adult ICU patients, regardless of MRSA colonization status. (Quality of evidence: MODERATE)

Unresolved issues	
**1**	Universal MRSA decolonization
**2**	Mupirocin and chlorhexidine resistance
**3**	MRSA-colonized HCP

Note. MRSA, methicillin-resistant *Staphylococcus aureus*; CDC, Centers for Disease Control and Prevention; WHO, World Health Organization; HCP, healthcare personnel.

### Essential practices

- Antimicrobial stewardship has been reclassified from an unresolved issue to an essential practice.
- Although contact precautions remain an essential practice, considerations have been provided for hospitals that have strong horizontal prevention measures and neither ongoing MRSA outbreaks nor high or increasing rates of MRSA infection or hospital-onset MRSA-positive cultures and that choose to modify the use of contact precautions for some or all MRSA-colonized or MRSA-infected patients.

### Additional approaches

- Active surveillance testing (AST) remains an additional practice, but specific recommendations, supporting data, and quality-of-evidence ratings for the use of AST in several specific patient populations have been added.
- Decolonization therapy for patients with MRSA colonization remains an additional practice, but specific recommendations, supporting data, and quality-of-evidence ratings for the use of universal or targeted decolonization in several specific patient populations have been added.

## Intended use

This document was developed following the process outlined in the *Handbook for SHEA-Sponsored Guidelines and Expert Guidance Documents.* ^2^ No guideline or expert guidance document can anticipate all clinical situations, and this document is not meant to be a substitute for individual clinical judgment by qualified professionals.

This document is based on a synthesis of evidence, theoretical rationale, current practices, practical considerations, writing-group consensus, and consideration of potential harm, where applicable. A summary recommendations is provided in Table 1.

## Methods

SHEA recruited 2 subject-matter experts in the prevention of MRSA to lead the panel of members representing the Compendium partnering organizations: SHEA, IDSA, APIC, AHA, and The Joint Commission, as well as the Centers for Disease Control and Prevention (CDC).

SHEA utilized a consultant medical librarian who worked with each panel to develop a comprehensive search strategy for PubMed and Embase (January 2012–July 2019, updated to August 2021). Article abstracts were reviewed by panel members in a double-blind fashion using the abstract management software Covidence (Melbourne, Australia) and were subsequently reviewed as full text. The Compendium Lead Authors group voted to update the literature findings, and the librarian reran the search to update it to August 2021. Panel members reviewed the abstracts of these articles via Covidence and incorporated relevant references.

Recommendations resulting from this literature review process were classified based on the quality of evidence and the balance between desirable and potential for undesirable effects of various interventions (Table 2). Panel members met via video conference to discuss literature findings; recommendations; quality of evidence for these recommendations; and classification as essential practices, additional approaches, or unresolved issues. Panel members reviewed and approved the document and its recommendations.

**Table 2.** Quality of Evidence

Quality of Evidence	
High	Highly confident that the true effect lies close to that of the estimated size and direction of the effect. Evidence is rated as “high” quality when there are a wide range of studies with no major limitations, there is little variation between studies, and the summary estimate has a narrow confidence interval.
Moderate	The true effect is likely to be close to the estimated size and direction of the effect, but there is a possibility that it is substantially different. Evidence is rated as “moderate” quality when there are only a few studies and some have limitations but not major flaws, there is some variation between studies, or the confidence interval of the summary estimate is wide.
Low	The true effect may be substantially different from the estimated size and direction of the effect. Evidence is rated as “low” quality when supporting studies have major flaws, there is important variation between studies, the confidence interval of the summary estimate is very wide, or there are no rigorous studies.

The Compendium Expert Panel, composed of members with broad healthcare epidemiology and infection prevention expertise, reviewed the draft manuscript after consensus had been reached by writing-panel members. Following review and approval by the Expert Panel, the 5 partnering organizations, stakeholder organizations, and the CDC reviewed the document. Prior to dissemination, the guidance document was reviewed and approved by the SHEA Guidelines Committee, the IDSA Standards and Practice Guidelines Committee, and the Boards of SHEA, IDSA, and APIC, as well as by the AHA and The Joint Commission.

All panel members complied with SHEA and IDSA policies on conflict-of-interest disclosure.

## Section 1: Rationale and statements of concern

### Burden of MRSA infection

1. HAIs caused by MRSA are common in acute-care facilities.
  - Worldwide, an estimated 15% of ICU infections are caused by *Staphylococcus aureus*, and nearly one-third of those (31%) are due to MRSA.^3^ In North America, an estimated 23% of ICU infections are caused by *S. aureus*, and nearly half of those (44%) are due to MRSA.
  - In the United States, *S. aureus* remains one of the most common pathogens associated with HAI.
    - Among the device-associated infections and surgical site infections (SSIs) reported to the CDC National Healthcare Safety Network (NHSN) between 2015 and 2017, *S. aureus* was the first and second most common pathogen reported in pediatric and adult infections, respectively.^4,5^
    - During this period, 48.4% of device-associated infections and 41.9% of SSIs caused by *S. aureus* were due to MRSA. Among device-associated *S. aureus* infections, rates of methicillin resistance ranged from 36.9% among possible ventilator-associated pneumonia (PVAP) to 51.7% among central-line–associated bloodstream infections (CLABSIs).^5^ Compared to data from 2009–2010, the proportions caused by MRSA are lower for each of these HAIs.^6^
    - A national study examining *S. aureus* bloodstream infections in the United States reported that the rate of hospital-onset MRSA bloodstream infections decreased 17% per year between 2012 and 2017.^7^
    - Although these findings suggest some success in preventing healthcare-associated MRSA transmission and infection, many patients and patient groups continue to be at risk. In fact, hospital-onset MRSA bloodstream infections increased 15% in US hospitals between 2019 and 2020 in association with the onset of the COVID-19 pandemic.^8^ This finding provides an important reminder of the importance of implementation of and adherence to preventive measures.
2. Outcomes associated with MRSA HAIs
  - MRSA infections are associated with significant morbidity and mortality.
  - An estimated 80,461 invasive MRSA infections occurred in the United States in 2011, with an all-cause in-hospital mortality rate of 14%.^9^
  - Another US study reported an unadjusted in-hospital mortality rate of 29% for hospital-onset MRSA bloodstream infections occurring between 2012 and 2017.^7^
  - A recent study using 2010–2014 data from the National Inpatient Sample from the Agency for Healthcare Research and Quality compared costs of hospitalization between MSSA and MRSA infections and noted that costs associated with MSSA infection approach those for MRSA infection. However, a higher adjusted mortality rate for MRSA-related hospitalizations was observed.^10^

### Risk factors for MRSA

1. MRSA HAI among colonized patients
  - A substantial proportion of colonized patients will subsequently develop a MRSA infection such as pneumonia, soft-tissue infection, or primary bloodstream infection.^11–16^ Among adults, this proportion has ranged from 9% to 33%.^17^
    - Risk of infection among those colonized is not limited to the period of concomitant hospitalization but persists beyond discharge. One study of persons in whom MRSA colonization had been identified during a previous hospital stay reported that the risk of developing a MRSA infection within 18 months of detection of MRSA colonization was 29%.^11^ Others have reported that among those who develop MRSA infections after discharge, these account for a substantial number of readmissions.^12^ A more recent study, in which individuals identified during hospitalization to be MRSA carriers were followed, found that 9% developed MRSA infection within 1 year and that 85% of those who developed MRSA infection required hospitalization.^17^
  - Among pediatric patients, 8.5% of children found to be colonized on admission subsequently developed a MRSA infection. Also, among patients who acquired MRSA colonization while being cared for in the pediatric intensive care unit, 47% subsequently developed MRSA infection.^16^
2. Risk factors for MRSA colonization and HAI
  - Risk factors for MRSA colonization include severe underlying illness or comorbid conditions, prolonged hospital stay, exposure to broad-spectrum antimicrobials, the presence of invasive devices such as central venous catheters, and frequent contact with the healthcare system or healthcare personnel (HCP).
  - Colonization pressure (the ratio of MRSA-carrier days to total patient days) has been identified as an independent risk factor for hospital-associated acquisition of MRSA.^18^
  - Community-associated MRSA (CA-MRSA) strains are a significant problem among persons without traditional healthcare-related risk factors^18–20^; however, transmission of CA-MRSA can and does occur in hospitals.^19–24^
    - In recent studies, an increasing proportion of hospital-onset invasive MRSA infections have been caused by community strains.^25^
    - Genomic studies suggest that there is an intermixing of community and hospital transmission networks for MRSA, underscoring that community factors should be an important consideration in determining MRSA risk.^26^
    - Estimates from the CDC Emerging Infections Program from 2011 to 2016 demonstrated the significant intersection of the opioid epidemic and invasive MRSA infections. Injection drug users were 16.3 times more likely to have an invasive MRSA infection than others.^27^
    - MRSA colonization and infection is occurring more frequently in those without classic risk factors. Therefore, community exposures (eg, injection drug use, correctional-facility exposure, crowding, and unstable housing) need to be considered as risk factors.^27–30^
3. Reservoir for MRSA transmission in acute-care facilities
  - In healthcare facilities, antimicrobial use provides a selective advantage for MRSA to survive.
  - The reservoir for MRSA in hospitals includes colonized or infected patients and HCP as well as contaminated objects within the patient care environment. Transmission is complex but occurs largely through patient-to-patient spread.
    - MRSA-colonized and MRSA-infected patients readily contaminate their environment, and HCP coming into contact with the patient or their environment readily contaminate their hands, clothing, and equipment.^31–43^
    - The risk for acquisition of MRSA is higher among hospital patients admitted to a room in which the previous occupant was colonized or infected with MRSA than among patients admitted into a room in which the previous patient was not colonized or infected with MRSA.^41,44^

## Section 2: Background on detection of MRSA

### Surveillance definitions for MRSA

1. Laboratory-identified event surveillance (ie, surveillance based on identification of MRSA laboratory results) and clinical infection surveillance are the 2 commonly used approaches for MRSA surveillance. These 2 surveillance strategies are not mutually exclusive and are often used in conjunction with one another.
  - Regardless of the type of MRSA surveillance selected for use, consistent application of the chosen surveillance definitions is necessary to generate reliable and accurate data that will allow detection of changes in the epidemiology of MRSA within the facility over time.
  - The CDC NHSN definitions for laboratory-based surveillance and infection surveillance are frequently used for MRSA surveillance.^45^ Because surveillance definitions are subject to change and refinement, users should always refer to source documents (eg, NHSN protocols) to determine currently recommended definitions.
2. Laboratory-identified event surveillance: The NSHN laboratory-identified event reporting definitions provide proxy measures of MRSA healthcare acquisition, exposure burden (colonization pressure or prevalence), and infection burden based solely on laboratory data and basic admission data (eg, date of admission, inpatient location).^45^
  - These definitions allow classification of clinical MRSA cultures as either healthcare-facility onset or community onset.
  - Similar definitions have also been published by SHEA and the Healthcare Infection Control Practices Advisory Committee.^46^
3. Clinical infection surveillance: Clinical infection surveillance can also be used to classify MRSA isolates as healthcare or community onset and to identify patients with specific types of healthcare-associated MRSA infection (eg, CLABSI or SSI).^45^
  - Unlike laboratory event–based definitions, which classify cultures based solely on the time of specimen collection relative to time of hospital admission, clinical infection surveillance definitions also include an evaluation of the patient’s clinical history and prior healthcare exposures.

### Surveillance methods for MRSA and detection of patients with MRSA

1. The reservoir for transmission of MRSA is largely composed of 2 groups of patients: those with clinical MRSA infection and a much larger group who are asymptomatic MRSA carriers. Various detection methods can be used to identify one or both groups.
  - Routine review of data from clinical specimens: Clinically infected patients and some asymptomatically colonized patients can be detected when MRSA is isolated from a clinical specimen obtained for clinical decision-making purposes.
  - Review of active surveillance testing (AST) data: AST for MRSA is defined as diagnostic testing performed to identify persons who are asymptomatic carriers of MRSA. AST is discussed in more detail in Section 4 and the Appendix.

## Section 3: Background on prevention of MRSA

### Summary of existing guidelines and recommendations

1. Several governmental, public health, and professional organizations have published evidence-based guidelines and/or policies for the prevention and control of MRSA.^47,48^ These guidelines provide similar recommendations, differing primarily on the emphasis placed on the use of AST to identify patients asymptomatically colonized with MRSA and in recommendations for routine decolonization of MRSA carriers.
2. IHI^49^ and APIC^50^ have developed practical suggestions for implementation and monitoring of several of the prevention measures specified in evidence-based guidelines.

### Infrastructure requirements

1. Infrastructure requirements of a MRSA prevention program include the following:
  - An infection prevention and control program that (1) is staffed by sufficient trained HCP to implement and sustain MRSA surveillance and prevention efforts without compromising other infection prevention and control activities and (2) has the authority to implement preventive measures.
  - Information technology systems that (1) can allow rapid notification of clinical staff and infection prevention and control HCP of new MRSA isolates, (2) can collect data needed for MRSA surveillance and outcome measure calculations, and (3) can identify MRSA-colonized patients upon readmission.
  - Sufficient supplies for hand hygiene, contact precautions (eg, gowns and gloves), environmental cleaning and disinfection, and other infection prevention interventions implemented as part of the facility’s MRSA control program.
  - An antimicrobial stewardship program is an important part of many quality and safety metrics, including MRSA prevention. The reader is referred to Barlam et al^51^ for a more detailed description of antimicrobial stewardship program infrastructure.
  - Resources to provide appropriate education and training to direct care and other HCP, patients, and visitors.
  - Adequate laboratory support: sufficient staffing and resources for routine clinical testing and for additional testing (ie, active surveillance) when necessary, and timely provision of relevant data to clinicians and the infection prevention program.
  - Leadership accountability and support in prioritizing resources needed to maintain a MRSA prevention program and implement effective interventions.

## Section 4: Recommended strategies to prevent MRSA

Recommendations are categorized as either (1) essential practices that should be adopted by all acute-care hospitals or (2) additional approaches that can be considered in locations and/or populations within hospitals when MRSA transmission is not controlled by essential practices. Essential practices include recommendations in which the potential to affect risk for transmission or infection of MRSA clearly outweighs the potential for undesirable effects. Additional approaches include recommendations in which the intervention is likely to reduce MRSA risk but there is concern about the risks for undesirable outcomes, recommendations for which the quality of evidence is low, recommendations in which cost-to-benefit ratio may be high, and recommendations in which evidence supports the impact of the intervention in select settings (eg, during outbreaks) or for select patient populations. Hospitals can prioritize their efforts by initially focusing on implementation of the prevention strategies listed as essential practices. If MRSA surveillance or other risk assessments suggest ongoing opportunities for improvement, hospitals should consider adopting some or all of the prevention approaches listed as additional approaches. These can be implemented in specific locations or patient populations or can be implemented hospital-wide, depending on outcome data, risk assessment, and/or local requirements. Each infection prevention recommendation has been given a quality-of-evidence grade (Table 2).

### Essential practices for preventing MRSA recommended for all acute-care hospitals

1. **Implement a MRSA monitoring program. (Quality of evidence: LOW)**
  - The MRSA monitoring program should do the following:
    - Identify any patient with a current or prior history of MRSA to ensure application of infection prevention strategies for these patients according to hospital policy (eg, contact precautions).
    - Provide a mechanism for tracking hospital-onset cases of MRSA for purposes of assessing transmission and infection and the need for response.
2. **Conduct a MRSA risk assessment. (Quality of evidence: LOW)**
  - The risk assessment should be attentive to 2 important factors: the opportunity for MRSA transmission and estimates of the facility-specific MRSA burden and rates of transmission and infection.
    - The opportunity for transmission is affected by the proportion of patients who are MRSA carriers (colonization prevalence) who serve as a reservoir for transmission. Estimates of facility-specific MRSA transmission and infection rates reflect the ability of the facility’s current activities to contain MRSA, regardless of the burden of MRSA that is imported into the facility.
    - Both colonization prevalence from sites performing active surveillance and rates of transmission and infection (eg, MRSA bloodstream infections, all MRSA-positive cultures) can be measured at either the total hospital level or for specific hospital units.
  - Findings from the risk assessment should be incorporated into the overall infection control program risk assessment and used to develop or refine mitigation strategies, surveillance, and goals based on the program’s prioritized risks.
  - Data used for initial and ongoing risk assessment can provide a baseline and can be used to monitor trends to inform the need for additional interventions. Metrics that might be used in the MRSA risk assessment are discussed in greater detail in Section 5 of this document.
3. **Promote compliance with CDC or World Health Organization (WHO) hand hygiene recommendations. (Quality of evidence: MODERATE)**
  - Hand hygiene is a fundamental strategy for the prevention of pathogen transmission in healthcare facilities.^17,52^
  - A common mode of transmission of MRSA to patients is by contact with contaminated hands of HCP, and some investigators have attributed reduced rates of MRSA among hospital inpatients in part to efforts made to improve hand hygiene practices of HCP.^53,54^
  - Promote patient hand hygiene.
4. **Use contact precautions for MRSA-colonized and MRSA-infected patients. (Quality of evidence: MODERATE). A facility that chooses or has already chosen to modify the use of contact precautions for some or all of these patients should conduct a MRSA-specific risk assessment to evaluate the facility for transmission risks and to assess the effectiveness of other MRSA risk mitigation strategies (eg, hand hygiene, cleaning and disinfection of the environment, single occupancy patient rooms) and should establish a process for ongoing monitoring, oversight, and risk assessment.**
  - Evidence for the use of contact precautions for MRSA-colonized and MRSA-infected patients
    - Studies have demonstrated that HCP interacting with MRSA-colonized or MRSA-infected patients often become contaminated with the organism.^42,43,55,56^
    - Similarly, studies in acute-care hospitals have demonstrated that surfaces and objects in the patient’s environment frequently and quickly become contaminated.^57–60^ Placing patients with MRSA colonization or infection under contact precautions may help reduce patient-to-patient spread of MRSA within the hospital.^61–64^
    - Several recent nonrandomized studies and reports support the use of contact precautions for MRSA-colonized and MRSA-infected patients.^56,64^ From 2005 to 2016, the incidence of hospital-onset MRSA bloodstream infections in the United States declined 74%.^7^ The reasons for this decline probably are multifactorial, but interventions to reduce MRSA transmission likely played a role. In 2007, the US Department of Veterans’ Affairs (VA) implemented a MRSA prevention bundle at VA acute-care hospitals nationwide. Introduction of this bundle, which included universal nasal surveillance for MRSA, contact precautions for MRSA carriers, hand hygiene, and increased institutional awareness of infection control, was associated with significant reductions in healthcare-associated MRSA infections and MRSA transmission in ICU and non-ICU settings.^64^ By 2017, hospital-onset MRSA infections at VA hospitals had declined 66% compared to baseline, while hospital-onset MSSA infections declined by only 19%.^65^ Decreases in MRSA infections at VA hospitals during this time were significantly higher among patients with negative MRSA admission screening tests compared to those with positive MRSA admission screening tests, suggesting that interventions to decrease transmission within hospitals played a large role in reducing MRSA infections. A mathematical modeling study published in 2021 of the VA MRSA prevention intervention estimated that contact precautions alone reduced MRSA transmission by 47%.^66^ A large cluster-randomized trial conducted in ICUs outside the VA system demonstrated significant reductions in MRSA transmission with the implementation of universal glove and gown use.^63^ In this trial, mathematical models estimated that universal glove and gown use was estimated to have reduced transmission by 44%.^56^
    - Based on a 2020 review of the current evidence, the CDC continues to recommend the use of contact precautions for MRSA colonized or infected patients^67^
    - During the COVID-19 pandemic, hospital-onset MRSA bloodstream infections increased nationally; however, whether declining use of contact precautions for MRSA-colonized or MRSA-infected patients played a significant role in this increase remains unknown.^68^
    - Studies have suggested that patients may be persistent MRSA carriers for prolonged periods (median duration in one study, 8.5 months).^69,70^ Use of contact precautions for patients with a history of MRSA is recommended.^67^ However, the appropriate duration of contact precautions necessary for patients with MRSA remains an unresolved issue. Further considerations for discontinuing contact precautions for patients with MRSA can be found in the SHEA Expert Guidance by Banach et al.^71^
  - Numerous studies have attempted to address whether contact precautions lead to an increase in adverse events.^72–74^ Some observational studies have shown an increase in adverse events including increased depression, anxiety, falls, electrolyte disorders, and decreased patient satisfaction.^74,75^ However, most of these studies did not control for comorbidity of patients and severity of illness of patients; thus, they suffer from confounding by indication. The only randomized trial to assess whether contact precautions lead to more adverse events showed a significantly lower frequency of HCP visits per hour (4.28 vs 5.24; *P* = .02) in ICUs using gowns and gloves for contact with all patients compared with control ICUs using gowns and gloves only for patients known to be colonized or infected with antimicrobial-resistant organisms and as otherwise required for CDC-defined contact precautions.^63^ The incidence of adverse events, though, was not significantly different between the 2 groups. In fact, rates of preventable, nonpreventable, severe, and nonsevere ICU adverse events were all nonsignificantly lower in the intervention group. Rates of hand hygiene on room exit were significantly higher in the universal glove-and-gown group. With randomized trials being a higher level of evidence than observational studies, current evidence does not indicate that contact precautions lead to an increase in adverse events.
  - Evidence on the impact of discontinuation of contact precautions for MRSA-colonized and MRSA-infected patients:
    - In recent years, several studies have sought to characterize the impact of discontinuing contact precautions for MRSA-colonized and MRSA-infected patients. Many of these studies have demonstrated that discontinuing contact precautions did not lead to an increase in HAIs.^76–78^ However, most were single-center, quasi-experimental studies that were underpowered and did not assess the effect of discontinuing contact precautions on MRSA acquisition or postdischarge MRSA infections. Thus, they were not designed to adequately detect the full impact of discontinuing contact precautions. Only 2 discontinuation studies used MRSA acquisition as an outcome.^79,80^ We acknowledge that, due to the large cost of performing cluster-randomized trials, no trial at present has evaluated contact precautions versus no contact precautions for MRSA. The closest study was the BUGG trial, which demonstrated significant reductions in MRSA acquisitions in ICUs that adopted universal gown-and-glove use.^56^
  - Considerations for facilities that choose to modify the use of contact precautions for some or all MRSA-colonized or MRSA-infected patients:
    - Hospitals should conduct a MRSA risk assessment based on internal infection rates, local epidemiology, hospital infrastructure (eg, proportion of non-private patient room) that may contribute to patient-to-patient transmission of MRSA if contact precautions are not used, and other factors. Please refer to Essential Practices recommendations 2. and 4.f.2 regarding use of a MRSA risk assessment and Section 5 for a list of metrics that can be used in the risk assessment.
      1. When making the decision to discontinue contact precautions for all or a subset of patients with MRSA, a facility should establish a policy and process that supports and communicates this change.
      2. At a minimum, a facility should provide guidance related to inclusion and exclusion criteria related to the process change; laboratory testing and surveillance strategies; implementation and communication; ongoing risk assessment; and oversight (eg, infection prevention committee) as appropriate.
    - Hospitals with ongoing MRSA outbreaks or with high or increasing rates of MRSA infection or hospital-onset MRSA-positive cultures* should not discontinue contact precautions for MRSA-colonized or MRSA-infected patients. *If active surveillance testing is used hospital-wide or in select situations, data regarding rates of acquisition of MRSA colonization may also be used in decisions to modify the use of contact precautions.
    - Based on the risk assessment, hospitals may choose to prioritize certain high-risk populations for which to continue contact precautions. High-risk populations identified may include the following:
      1. ICU patients
      2. NICU patients
      3. Burn-unit patients
      4. Dialysis patients
      5. Transplant and other specialty units with immunocompromised patients
      6. Patients with indwelling devices such as central venous catheters
      7. Patients with active infections, particularly those with uncontained wounds or secretions
      8. Residents of long-term acute-care hospitals
      9. Residents of long-term care facilities
    - Hospitals that choose to modify the use of contact precautions for some or all MRSA-colonized or MRSA-infected patients should, at a minimum, have strong horizontal prevention practices in place and demonstrate high adherence to these mitigation strategies. These practices may include audits, rounding, and teams to address the following:
      1. Hand hygiene
      2. Standard precautions
      3. Environmental cleaning and disinfection
      4. PPE adherence and discontinuation of extended use and reuse of gowns and gloves
      5. CLABSI prevention
      6. SSI prevention
  - Hospitals that choose to modify the use of contact precautions for some or all MRSA-colonized or MRSA-infected patients should consider implementing a MRSA decolonization program for certain high-risk groups or high-risk settings (eg, ICUs). (See decolonization recommendations in the Additional Approaches section.)
  - Hospitals that choose to modify the use of contact precautions for some or all MRSA-colonized or MRSA-infected patients should monitor key metrics (see 4.f.2) and reconsider the use of contact precautions if an outbreak occurs or if MRSA rates increase.
    - Establish appropriate metrics that capture changes in rates of MRSA infection or transmission. Incorporate these metrics in the ongoing risk assessment and make adjustments to the use of contact precautions or other infection prevention strategies when appropriate. Note: These metrics may be underpowered and limited in their ability able to identify all downstream effects of changes to the use of contact precautions.
    - Possible key metrics to monitor include the following:
      1. MRSA clinical culture positivity rates
      2. Hand hygiene compliance
      3. Compliance with hospital designated decolonization protocols including chlorhexidine bathing and intranasal treatment (eg, mupirocin)
      4. Hospital-onset MRSA infections, including device-associated infections, procedure-associated infections such as SSIs, bloodstream infections, and other infection types such as pneumonia or skin and soft tissue as appropriate based on historical data
      5. MRSA acquisition rates if active surveillance testing is in place (see active surveillance testing recommendations in Section 5, Additional Approaches for Preventing MRSA Infection)
      6. Rates of admission with new MRSA infection or colonization (among persons without prior history of MRSA colonization or infection) within 30–90 days of prior hospital discharge
        - This metric is intended to identify patients who may have acquired MRSA during a recent hospital admission. Studies have demonstrated that prior hospitalization is a common risk factor for non–hemodialysis-related healthcare-associated community-onset MRSA infection, with the majority occurring within 12 weeks of a prior hospital admission.^9^
5. **Ensure cleaning and disinfection of equipment and the environment. (Quality of evidence: MODERATE)**
  - MRSA contaminates the patient environment (eg, overbed tables, bedrails, furniture, sinks, floors) and patient care equipment (eg, stethoscopes, blood pressure cuffs, etc).^81^ MRSA contamination on surfaces around the patient zone varies in bioburden concentration.
  - Exposure to this contaminated environment has been associated with acquisition of MRSA.^41^ Improvements in environmental cleaning have been associated with reductions in MRSA acquisition among patients admitted to rooms in which the previous occupant was colonized or infected with MRSA.^82^
  - Cleaning and disinfection are horizontal infection practices that can prevent transmission of multiple pathogens.
  - Objective monitoring of the thoroughness of cleaning and disinfection using direct observation, fluorescent marking systems, and/or ATP detection systems with feedback of monitoring results to personnel responsible for cleaning has been associated with improvements in environmental cleaning and disinfection in healthcare settings.
6. **Implement a laboratory-based alert system that notifies HCP of new MRSA-colonized or MRSA-infected patients in a timely manner. (Quality of evidence: LOW)**
  - Timely notification of new MRSA-positive test results to clinical caregivers and infection preventionists facilitates rapid implementation of contact precautions and other interventions (eg, treatment of infection) as appropriate according to facility policy, assessment of risk, and timely surveillance for HAIs.
7. **Implement an alert system that identifies readmitted or transferred MRSA-colonized or MRSA-infected patients. (Quality of evidence: LOW)**
  - An alert system allows information regarding the MRSA status of the patient to be available at the first point of contact (eg, emergency department arrival, presentation to admitting department), prior to bed assignment, to promptly initiate appropriate control measures and minimize opportunities for transmission.
  - Alerts facilitate early prevention interventions within the continuity of care, such as internal transfers between inpatient units or interfacility transfers managed via regional patient transfer centers.
  - Communication at the time of procedure scheduling and verbal hand-off safety practices (eg, SBAR—situation, background, assessment, recommendation) allows for planning and continuity of prevention activities at the time of patient transport and in the receiving service department (ie, imaging, cardiac catheterization, etc).
8. **Provide MRSA data and outcome measures to key stakeholders, including senior leadership, physicians, nursing staff, and others. (Quality of evidence: LOW)**
  - Provision of MRSA data and other information related to the activities of the MRSA prevention program to key stakeholders on a regular and frequent basis may optimize focus on MRSA prevention efforts, substantiate requests for resources, and increase engagement in the MRSA prevention program. (See Section 5 for suggested metrics for assessment of the MRSA prevention program.)
9. **Educate healthcare personnel (HCP) about MRSA. (Quality of evidence: LOW)**
  - Several key components of an effective MRSA prevention program involve modification of HCP behavior (eg, hand hygiene, contact precautions, environmental cleaning, and disinfection).
  - HCP should be educated about their role in MRSA prevention and other MRSA-related topics as appropriate.
10. **Educate patients and families about MRSA. (Quality of evidence: LOW)**
  - Patients and their families should be educated regarding the importance of hand hygiene and respiratory etiquette to reduce the risk of spread of MRSA and other pathogens during the hospital stay.
  - Patients who are colonized or infected with MRSA and their families should be educated about MRSA and what they can do to reduce the risk of infection and transmission.
11. **Implement an antimicrobial stewardship program. (Quality of evidence: LOW)**
  - Receipt of antibiotics without MRSA activity has been associated with significant increases in the intranasal burden of MRSA.^83^ Thus, receipt of such antibiotics may increase the risk of infection in the colonized person and/or increase risk of transmission to others.
  - However, the association between antimicrobial stewardship interventions and rates of MRSA infection and colonization has varied among studies. Of 3 recent systematic reviews and/or meta-analyses, 2 found an association between implementation of antimicrobial stewardship interventions and a decreased incidence of MRSA infection and/or colonization.^84–86^
  - The quality of evidence for antimicrobial stewardship as a component of a MRSA prevention program is low (eg, mostly single-center, nonrandomized, uncontrolled studies). However, a theoretical rationale and some evidence of benefit do exist, and no evidence of harm has been reported. In addition, benefits of antimicrobial stewardship have been established for other important outcomes (eg, *C. difficile* prevention).
  - Please refer to the “Compendium of Strategies to Prevent Surgical Site Infections in Acute Care Hospitals: 2022 Update”^87^ and current guidelines for surgical antibiotic prophylaxis^88^ for recommendations regarding surgical antibiotic prophylaxis among patients known to be colonized with MRSA.

## Additional approaches for preventing MRSA infection

### Active surveillance testing (AST)

Active surveillance testing is based on the premise that clinical cultures identify only a small proportion of hospital patients who are colonized with MRSA and that these asymptomatic carriers serve as a substantial reservoir for person-to-person transmission of MRSA in the acute-care hospital. Studies have reported that clinical cultures alone may underestimate the overall hospital prevalence of MRSA by as much as 85% and the monthly average prevalence of MRSA in ICUs by 18.6%–63.5%.^24,89,90^ AST is used to identify these asymptomatic MRSA carriers so that additional infection control measures (eg, contact precautions, decolonization) can be put into place to decrease the risk of transmission to other patients and HCP and/or to decrease the risk of infection to carriers themselves (decolonization). AST is also used as part of antibiotic stewardship to reduce vancomycin usage,^91,92^ to clear contact precautions,^93,94^ and as part of implementing postdischarge interventions.^17,95,96^

1. **Implement a MRSA active surveillance testing (AST) program for select patient populations as part of a multifaceted strategy to control and prevent MRSA. (Quality of evidence: MODERATE).** ^97^ Recommendations for specific populations may have different evidence ratings.
2. **Active surveillance for MRSA in conjunction with decolonization can be performed in targeted populations prior to surgery to prevent postsurgical MRSA infection. (Quality of evidence: MODERATE)**
  - A large meta-analysis demonstrated a reduction in MRSA surgical site infection (SSI) when active surveillance was coupled with targeted nasal decolonization of MRSA carriers prior to undergoing surgery with hardware.^98^ Several other studies, including large clinical trials, have demonstrated a similar reduction in both SSI and nosocomial disease when employing *S. aureus* active surveillance and targeted decolonization of carriers. (See MRSA Decolonization, recommendation 2, in the Additional Approaches section.)
  - Please refer to the “Compendium of Strategies to Prevent Surgical Site Infections in Acute Care Hospitals: 2022 Update”^87^ for recommendations regarding active surveillance and decolonization for organisms other than MRSA (eg, all *S. aureus*).
3. **Active surveillance with contact precautions is inferior to universal decolonization for reduction of MRSA clinical isolates in adult ICUs. (Quality of evidence: HIGH)**
  - A 43-hospital cluster-randomized trial in ICUs (REDUCE MRSA Trial)^99^ directly compared (1) active surveillance for MRSA coupled with contact precautions, (2) active surveillance for MRSA coupled with contact precautions and targeted decolonization, and (3) stopping active surveillance, continuing contact precautions for known MRSA carriers, and performing universal decolonization for all ICU patients. Universal decolonization with chlorhexidine bathing and nasal mupirocin was superior to the other arms, resulting in a 37% reduction in MRSA clinical isolates (from 3.4 per 1,000 ICU days to 2.1 per 1,000 ICU days) and a 44% reduction in all-cause bloodstream infections (6.1 per 1,000 ICU days to 3.6 per 1,000 ICU days). Universal decolonization should be pursued in lieu of targeted actions informed by active surveillance for the purpose of reducing MRSA.
4. **Hospital-wide active surveillance for MRSA can be used in conjunction with contact precautions to reduce the incidence of MRSA infection. (Quality of evidence: MODERATE)**
  - Most hospitals across the United States do not perform active surveillance for all patients.^100^ However, between 2007 and the beginning of the COVID-19 pandemic, US Department of Veterans’ Affairs (VA) acute-care hospitals conducted hospital-wide active surveillance. In 2007, VA acute-care hospitals nationwide launched a MRSA control program that included universal nasal active surveillance for MRSA, contact precautions for MRSA carriers, hand hygiene, and increased institutional awareness of infection control. This program resulted in significant reductions in healthcare-associated MRSA infections and MRSA transmission in ICU and non-ICU settings.^64^ By 2017, hospital-onset MRSA infections at VA hospitals had declined 66% compared to baseline, and hospital-onset MSSA infections had declined 19%.^65^ Questions have arisen regarding what aspects of the VA policy led to the decline, especially relative to active surveillance. Questions have also been raised about the generalizability of findings at VA acute-care hospitals to other hospitals.^101^ Hospitals that do not want to conduct whole-hospital active surveillance should consider instituting a more targeted policy based on high-risk patients or high-risk encounters.^42,43^ In addition, hospitals should consider using their baseline risk assessment and additional MRSA monitoring and assessments to help guide their decision making.^102^ (See the Risk Assessment and Contact Precautions recommendations in the Essential Approaches section above and Section 5 “Performance Measures” below.) Active surveillance testing has cost implications. Cost and yield considerations should be used to help guide cost-effective policies to attain reductions in MRSA transmission and disease.^103–105^
5. **Active surveillance can be performed in the setting of a MRSA outbreak or evidence of ongoing transmission of MRSA within a unit as part of a multifaceted strategy to halt transmission. (Quality of evidence: MODERATE).**
  - During outbreaks, serial (eg, weekly until outbreak is over) AST can provide important information about the scope of the outbreak, and AST helps identify new cases to enable communication and response (eg, contact precautions, decolonization).
  - See the Decolonization recommendations below for discussion of components of a multimodal strategy.
  - The CDC 2020 NICU guidelines provide information regarding application of this recommendation in the neonatal ICU.^48^
  - See the “Screen HCP for MRSA infection or colonization” recommendation below for additional discussion regarding use of AST for HCP.

### Screen HCP for MRSA infection or colonization

1. **Screen HCP for MRSA infection or colonization if they are epidemiologically linked to a cluster of MRSA infections. (Quality of evidence: LOW)**
  - HCP can become transiently or persistently colonized with MRSA and can be the source of hospital outbreaks.
    - Routine screening of HCP for MRSA is not currently recommended in the endemic setting.^106^
    - Screening of HCP can be an important component of an outbreak investigation if HCP have been epidemiologically linked to a clonal cluster of MRSA cases or if there is evidence of ongoing transmission despite comprehensive implementation of basic MRSA control measures.^106^
    - See MRSA decolonization below and Section 6: Implementation Strategies for discussion of targeted decolonization therapy regimens that can be used for the treatment of MRSA-colonized HCP.

### MRSA decolonization

MRSA decolonization therapy most commonly refers to the administration of topical antimicrobial or antiseptic agents for the purpose of eradicating or suppressing the carrier state and ultimately reducing clinical infection. MRSA decolonization can be targeted to MRSA carriers or applied universally to populations deemed at high risk for infection. For the purpose of this document, MRSA decolonization is considered to be intranasal antimicrobial and/or antiseptic treatment with chlorhexidine (CHG) skin antisepsis.

Because MRSA carriage is the strongest predictor of subsequent MRSA infection, decolonizing carriers is important if MRSA prevalence or disease is a target for improvement. Intranasal treatment is necessary to eliminate MRSA in the nose, which is recognized as a primary carriage site. Clearance of the nasal reservoir has been shown to be both necessary and sufficient for infection reduction among *S. aureus* carriers.^107–110^ A discussion of agents that have been used for nasal decolonization is provided in the Appendix. MRSA may also contaminate and/or colonize skin sites, most commonly axilla and groin, although other skin sites may also harbor MRSA. Skin antisepsis is often used during decolonization and for source control of MRSA. Finally, although nasal eradication of MRSA is a necessary component to prevent infection in MRSA carriers, some evidence indicates that skin antisepsis alone may reduce MRSA transmission to others in ICUs.^52^ Hospitals may choose to use a CHG-only decolonization strategy to target other pathogens or reduce bloodstream infections, but if the goal is to reduce MRSA, then nasal decolonization may be necessary to optimize the likelihood of success.

Several randomized clinical trials (discussed below) have shown that decolonization significantly reduces MRSA carriage, transmission, and subsequent infection in patients known to carry MRSA or to be at risk of MRSA acquisition and/or infection. These are discussed below within the specific recommendations.

*S. aureus* outcomes identified through the literature review for MRSA outcomes have been described due to the relevant interest for healthcare-associated infection (HAI) reduction from *S. aureus* regardless of susceptibility pattern, but the recommendations are based on available evidence to reduce MRSA.

Few studies of high-quality evidence have evaluated MRSA outcomes in children. Most data supporting the recommendations below have been generated in adult patient populations. When available, pediatric data are noted.

Complications of decolonization therapy are rare and generally mild; however, hospitals should be aware of potential adverse effects, such as drug-related toxicities and development of resistance (eg, mupirocin) or reduced susceptibility (eg, chlorhexidine) to the agents used, when considering the potential benefits and risks of implementing a MRSA or *S. aureus* decolonization program.^111–113^

1. **Use universal decolonization (daily CHG bathing plus 5 days of nasal decolonization) for all patients in adult ICUs to reduce endemic MRSA clinical cultures. (Quality of evidence: HIGH)**
  - The previously described REDUCE MRSA Trial demonstrated that universal decolonization of ICU patients with daily CHG bathing and 5 days of twice-daily mupirocin was superior to screening and targeted decolonization as well as to screening and targeted contact precautions for prevention of MRSA-positive clinical isolates and all-cause bloodstream infection.^99^ (See Section 4 Additional Approaches for Preventing MRSA, Active Surveillance Testing recommendation.) For determining the applicability of this regimen to hospitals, trial benefit occurred at fairly low endemic levels of >3 MRSA clinical cultures per 1,000 ICU days. This approach has been demonstrated to be cost-effective, including sparing the cost of screening.^103,114^
  - Climo et al^115^ reported that universal CHG alone in adult ICUs reduced bloodstream infections by 28% and reduced the composite of MRSA and vancomycin-resistant enterococcal (VRE) acquisition by 23%. Derde et al (2013)^52^ also demonstrated that CHG bathing decreased MRSA acquisition in ICU standardization phases leading up to an RCT that showed no benefit of either conventional or rapid PCR MRSA screening and isolation over high compliance hand hygiene and universal CHG bathing.
  - Even though universal CHG alone does not decolonize carriers, it is effective in reducing transmission of MRSA from carriers to noncarriers in ICUs. Thus, for the purpose of optimally addressing MRSA, the combined effects of mupirocin plus universal CHG are recommended.
  - Finally, although universal decolonization has been found to be superior to screening and targeted decolonization, hospitals may have other reasons for screening patients for MRSA. These may include outbreak response, desire for surveillance data, desire to implement contact precautions for known carriers, and clinical reasons related to restricting empiric anti-MRSA therapy or preoperative vancomycin prophylaxis to known MRSA carriers.
2. **Perform preoperative nares screening with targeted use of CHG and nasal decolonization in MRSA carriers to reduce MRSA SSI in surgical procedures involving implantation of hardware. (Quality of evidence: MODERATE)**
  - Note that decolonization can be applied universally as an alternative.
  - Preoperative targeted screening and decolonization of *S. aureus* carriers is commonly performed for surgical procedures involving the placement of hardware to reduce SSI. Although most studies have evaluated *S. aureus* outcomes and are not specific to MRSA, a large meta-analysis of RCTs and other studies involving surgeries with hardware similarly found that targeted or universal nasal decolonization reduced *S. aureus* SSI and that nasal decolonization of MRSA carriers reduced MRSA SSI.^116^
  - *S. aureus* outcomes were not the target of this guidance document or its search strategy. Nevertheless, we highlight some the *S. aureus* evidence here because MRSA is a subset of *S. aureus.* In a large, 20-hospital, interventional cohort study of cardiac, hip, and knee surgeries, targeted nasal decolonization reduced *S. aureus* SSI.^116^ Additionally, in a post-hoc analysis of a single-center RCT of 1,697 patients undergoing arthroplasty or spinal fusion, universal nasal 5% povidone-iodine was superior to universal 2% mupirocin for *S. aureus* deep SSI.^117^ Universal nasal decolonization without nasal screening can be employed for pragmatic reasons to spare the logistics for screening for *S. aureus* or MRSA. Use of povidone-iodine may also be chosen for pragmatic reasons because it does not require a prescription, including prescription-related transportation needs or insurance copays that may affect patient adherence.
  - Please refer to the “Compendium of Strategies to Prevent Surgical Site Infections in Acute Care Hospitals: 2022 Update”^87^ for recommendations regarding decolonization for organisms other than MRSA.
3. **Screen for MRSA and provide targeted decolonization with CHG bathing and nasal decolonization to MRSA carriers in surgical units to reduce postoperative MRSA inpatient infections. (Quality of evidence: MODERATE)**
  - Note that decolonization can be applied universally as an alternative.
  - In a multinational trial of 33 surgical units in 10 hospitals involving 126,750 admissions, an intervention of enhanced hand hygiene plus universal screening and targeted decolonization of MRSA carriers reduced MRSA clinical cultures by 12% per month. In a secondary analysis of clean surgical patients, universal screening and targeted decolonization of MRSA carriers reduced MRSA clinical cultures by 15% per month and MRSA infections by 17% per month.^118^
  - *S. aureus* outcomes were not the target of this guidance document nor its search strategy. Nevertheless, we highlight the key *S. aureus* evidence here because MRSA is a subset of *S. aureus.* In an RCT of 1,000 mostly surgical patients that evaluated universal inpatient screening for *S. aureus* and CHG and mupirocin for identified carriers, a significant 58% reduction was achieved in inpatient *S. aureus* infection among carriers.^119^ In addition, decolonization can reduce postsurgical inpatient infections beyond SSI. The Mupirocin and the Risk of *Staphylococcus aureus* (MARS) Study^120^ was a 3,864-person RCT of the addition of mupirocin to preoperative chlorhexidine (CHG) for *S. aureus* carriers undergoing a variety of surgical procedures (ie, general, gynecologic, neurologic, oncologic, and cardiothoracic surgery) with and without hardware. This mupirocin addition significantly decreased nosocomial *S. aureus* infections by 51% among *S. aureus* carriers, although it did not significantly reduce *S. aureus* SSIs.
4. **Provide CHG bathing plus nasal decolonization to known MRSA carriers outside the ICU with medical devices, specifically central lines, midline catheters, and lumbar drains, to reduce MRSA-positive clinical cultures. (Quality of evidence: MODERATE)**
  - The Active Bathing to Eliminate Infection (ABATE Infection) Trial^121^ was a 53-hospital cluster-randomized trial involving nearly 340,000 patients comparing routine care to universal decolonization with CHG bathing plus targeted nasal mupirocin for known MRSA carriers. Active screening was not a component of this trial. No overall reduction in the composite outcome of MRSA or VRE carriage, nor all-cause bloodstream infections was detected. However, in a post-hoc analysis, non-ICU patients with medical devices had a significant 37% reduction in MRSA and VRE and a significant 32% reduction in all-cause bloodstream infections. Patients with medical devices (specifically, central lines, midlines, and lumbar drains) were only 10% of inpatients, but they had 37% of MRSA and VRE cultures and 56% of all-cause bloodstream infections.
5. **Consider postdischarge decolonization of MRSA carriers to reduce postdischarge MRSA infections and readmission. (Quality of evidence: HIGH).**
  - The Changing Lives by Eradicating Antibiotic Resistance (CLEAR) Trial^17^ was an RCT to decrease postdischarge infections in MRSA carriers comparing routine care to postdischarge decolonization (CHG bathing, CHG mouthwash, nasal mupirocin) given for 5 days twice monthly for 6 months. The trial involved 2,121 MRSA carriers. Decolonization significantly reduced MRSA infection (most requiring rehospitalization) by 30% in the 1-year follow-up period, with a number needed to treat of 30. The impact of a shorter duration of decolonization is not known, but the risk of postdischarge infection was higher with closer proximity to discharge.
  - Postdischarge decolonization was first systematically performed by the Dutch Search and Destroy program to decolonize MRSA carriers to prevent infection.^122^ These postdischarge efforts require coordination and investment uncommonly adopted by hospitals. Because population-based medicine continues to be a goal for HAI prevention across the continuum of care, assessments of pragmatic implementation and adherence need to be addressed.
6. **Neonatal ICUs should consider targeted or universal decolonization during times of above-average MRSA infection rates or targeted decolonization for patients at high risk of MRSA infection (eg, low birth weight, indwelling devices, or prior to high-risk surgeries). (Quality of evidence: MODERATE)**
  - *S. aureus* is a leading cause of HAI in neonatal intensive care units (NICUs). Neonates in the NICU, especially low-birthweight neonates, are at high risk of invasive *S. aureus* disease.^123^ Because most neonates have never left the hospital, neonates usually develop MRSA colonization or infection as a result of hospital-based transmission. Neonates acquire MRSA from colonized parents, HCP, or the environment. MRSA is the most commonly reported cause of NICU outbreaks,^124^ so when neonates in the NICU are identified with a hospital-onset MRSA infection, further assessment is warranted to identify an ongoing cluster of transmission.
    - MRSA colonization is an important risk factor for subsequent infection in this population. Quasi-experimental studies have shown that decolonization can reduce MRSA infections during endemic and outbreak settings.^48,125^
    - Targeted and universal decolonization approaches have both been successfully used to reduce MRSA in this population.^126–128^ Decolonization reduces MRSA colonization, acquisition and infection in neonates.^129^
    - Decolonization also reduces MSSA colonization and infections in this population.^130–132^
    - Mupirocin and chlorhexidine are the most commonly used decolonization agents in NICUs. In a recent RCT, 66 infants were assigned to intranasal mupirocin, and no product-related moderate, serious, or severe adverse events occurred.^132^ Chlorhexidine has been safely used in neonates, but due to potential for skin irritation and systemic absorption, it should be used with caution in premature infants.^133^ The US Food and Drug Administration notes that chlorhexidine should be “used with care in premature infants or infants under 2 months of age.”^134^ Chlorhexidine is used widely in NICUs and its use increased from 59% in 2009 to 86% in 2015 in a survey of US NICUs.^135,136^ Chlorhexidine-associated adverse events are infrequent, but many NICUs limit chlorhexidine use, especially in preterm infants within the first month of life.^125,135^
    - In addition to HCP and the environment, parents can be an important reservoir for *S. aureus* and can expose their neonates in the NICU. The TREAT PARENTS trial showed that decolonizing parents with intranasal mupirocin and topical chlorhexidine gluconate baths reduced transmission of MRSA and MSSA to neonates in the NICU.^137^
7. **Burn units should consider targeted or universal decolonization during times of above-average MRSA infection rates. (Quality of evidence: Moderate)**
  - Higher quality evidence is needed to support a recommendation for routine decolonization of burn patients (unresolved issue).
  - Burn patients are at high risk of MRSA acquisition and infection.
  - Quasi-experimental studies have shown that decolonization can reduce MRSA infections. Decolonization strategies have included universal intranasal mupirocin with chlorhexidine antisepsis, universal decolonization using mupirocin and daily hypochlorous acid solution, and octenidine antisepsis for intact skin and nasal mucosa.^138–141^
  - Given inconsistent results on the safety of antiseptics to interfere with wound healing, the role of topical antisepsis in this population for MRSA prevention must carefully balance the risk of toxicity and benefit of preventing MRSA infections. Therefore, the decision to implement targeted or universal decolonization in burn patients should be guided by a local risk assessment of MRSA incidence.
8. **Consider targeted or universal decolonization of hemodialysis patients. (Quality of evidence: MODERATE)** Higher-quality evidence is needed to support a recommendation for routine decolonization of dialysis patients.
  - MRSA bloodstream infections complicate care of hemodialysis patients. MRSA colonization predisposes individuals to subsequent MRSA infections, and hemodialysis patients have one of the highest risks of MRSA invasive disease, with a risk of 45 per 1,000 patients, which as 100-fold higher than that of the average population.^142,143^
  - A systematic review and meta-analysis found that intranasal mupirocin with chlorhexidine body washes can eradicate MRSA carriage in hemodialysis patients.^144^Data are not available demonstrating effectiveness of decolonization on reducing MRSA infections. However, a separate systematic review and meta-analysis found an 82% reduction in the risk of *S. aureus* bacteremia, comparing those who did and did not receive mupirocin.^145^
9. **Decolonization should be strongly considered as part of a multimodal approach to control MRSA outbreaks. (Quality of evidence: MODERATE)**
  - Although no clinical trials have tested strategies to control MRSA outbreaks, many quasi-experimental studies have demonstrated successful outbreak control that includes MRSA decolonization as part of a multimodal approach to reduce MRSA transmission and infection.
  - In outbreak situations, decolonization can protect colonized individuals from infection and reduce colonization pressure that may promote transmission.
    - Intranasal therapy reduces infection risk for individual patients.
    - Topical skin decontamination reduces bioburden and helps reduce organism transmission.
  - Decolonization can be implemented universally or in combination with AST
    - In an outbreak setting, active surveillance cultures can help measure the extent of organism spread in the unit and provide organisms for strain typing.
    - In addition to identifying patients as a reservoir for propagating outbreaks, successful outbreak control may involve screening HCP to detect reservoirs, especially in high-risk units like the neonatal ICU and burn units.^146^ HCP have been implicated as reservoirs for MRSA transmission during adult hospital unit and NICU outbreaks and during times of ongoing clonal transmission.^147,148^ After implementation and failure of other basic MRSA prevention and control measures (eg, hand hygiene, contact precautions, enhanced environmental cleaning, screening and decolonizing neonates), screening and decolonizing HCWs has helped successfully control MRSA outbreaks in adult units and NICUs.^149–151^

### Universal use of gowns and gloves

1. **Use gowns and gloves when providing care to or entering the room of all adult ICU patients, regardless of MRSA colonization status. (Quality of evidence: MODERATE)**
  - A cluster-randomized trial conducted in 20 adult medical and surgical ICUs compared the effect of universal glove and gown use for all patient contact and when entering any patient room with standard practice (ie, the use of gowns and gloves only for patients known to be infected or colonized with antimicrobial-resistant organisms) on the rate of acquisition of antimicrobial-resistant gram-positive organisms and healthcare-associated infections.^63^ Although the investigators found no difference in the primary outcome of acquisition of either MRSA or VRE, there was a significantly greater relative reduction in the prespecified secondary outcome of MRSA acquisition in intervention units compared to control units (40.2% vs 15%; *P* = .046).
    - On intervention units, contamination of HCW clothing was 70% lower during the intervention period than during standard practice in the postintervention period (7.1% vs 23%; OR, 0.3; 95% CI, 0.2–0.6).^152^ In addition to the use of gowns and gloves, a lower frequency of HCP visits (4.28 vs 5.24 per hour; *P* = .02) and higher hand-hygiene compliance (78.3% vs 62.9% upon exit; *P* = .02) in the intervention arm compared to the control arm may have played a role in the observed difference in MRSA acquisition between the 2 groups. In subsequent mathematical modeling, the decrease in MRSA acquisition was found to be primarily due to the gown-and-glove use intervention, with additional but smaller effects from improved hand hygiene and lower HCP–patient contact rates.^56^
    - In a subsequent secondary analysis of data from this trial, the intervention was associated with a nonsignificant decrease in acquisition of antibiotic-resistant gram-negative bacteria (rate ratio, 0.90; 95% CI, 0.71–1.12).^153^ This finding suggests that universal gown-and-glove use when providing care in adult ICUs may provide benefits in addition to the potential to reduce MRSA transmission.

### Unresolved issues

Several unresolved issues remain related to MRSA and its transmission. A full discussion of these issues is beyond the scope of this document, but a brief mention of some of these important topics is worthwhile.

1. Universal MRSA decolonization
  - Additional study is needed to determine the incremental benefit of the addition of mupirocin to daily chlorhexidine bathing in the adult ICU because the REDUCE MRSA study used both mupirocin and CHG for their decolonization arm.^99^
  - Additional study is needed to evaluate the role of routine universal decolonization of NICU patients.
2. Mupirocin and chlorhexidine resistance: The risk for development of resistance to mupirocin and/or chlorhexidine as they become more widely used is currently unknown, although some centers have reported increased rates of resistance.
  - Chlorhexidine: Although some published data have demonstrated reduced susceptibility in vitro to chlorhexidine among staphylococci by at least 2 mechanisms of resistance, the definitions used in these studies often use an MIC threshold far below standard CHG applications (eg, often an MIC of 8 µg/mL is used to define “resistance,” even though 2% CHG applies 20,000 µg/mL to the skin). Clinical trials have evaluated, but have not identified, the emergence of resistance to CHG.^115,154^
  - Mupirocin resistance has been studied extensively; however, the ability of hospital laboratories to provide mupirocin resistance data is limited.
    - Mupirocin resistance is phenotypically categorized into 2 levels based on the minimum inhibitory concentration (MIC). Low-level resistance (MICs of 8–256 mg/mL), and high-level resistance (MICs > 512 mg/mL).^155^ The molecular mechanism of low-level mupirocin resistance involves point mutations and is mediated by plasmid encoded genes in high-level mupirocin.
    - A recent meta-analysis described a global increase in the prevalence high-level mupirocin resistance among clinical *S. aureus* isolates over time. Because mupirocin remains the most effective antibiotic for MSSA and MRSA decolonization, a reduction in its effectiveness presents a risk.^156^
    - Emergence of mupirocin resistance following increased use has not been reported consistently. The use of universal ICU decolonization with mupirocin in the REDUCE MRSA Trial was not associated with emergence of mupirocin resistance when evaluating thousands of MRSA isolates from the trial.^154^ Additional studies of mupirocin resistance have been hampered by a lack of availability of routine susceptibility testing in most hospital laboratories. Large-scale studies on decolonization failure associated with increased mupirocin use are needed to provide an understanding of the risk.
3. MRSA-colonized HCP: The optimal use of AST to identify asymptomatic carriage of MRSA among HCP and the optimal management (eg, decolonization therapy, follow-up monitoring) of MRSA-colonized HCP have not been definitively determined.

## Section 5: Performance measures

### Internal reporting

The performance measures described here are intended to support internal hospital quality-improvement efforts and do not necessarily address external reporting requirements. The process and outcome measures suggested here are derived from published guidelines and other relevant literature. A more detailed description of outcome measures that may be useful for MRSA transmission and infection prevention programs is available in a position paper published in 2008 by SHEA and HICPAC.^46^

### Process measures

Process measures can be used to assess compliance with various components of a MRSA prevention program. Such measures may include compliance with essential practices, such as hand hygiene and contact precautions (eg, use of gown and gloves), as well as compliance with additional approaches that have been implemented by the hospital (eg, daily bathing with chlorhexidine and/or AST).

### Outcome measures

In 2008, SHEA and the HICPAC published recommendations for monitoring multidrug-resistant organisms (MDROs) in healthcare settings.^46^ These recommendations are applicable to MRSA as well as other MDROs. That position paper describes the following MRSA outcome measures.

- Basic outcome measures for all acute-care hospitals
  1. MRSA-specific line lists (eg, electronic databases) for tracking patients who have MRSA;
  2. Annual antibiograms for monitoring antimicrobial susceptibility patterns (eg, rates of methicillin resistance) among isolates recovered from patients;
  3. Estimates of the MRSA infection burden that use objective, laboratory-based metrics such as the incidence (or incidence density) of hospital-onset MRSA bacteremia; and
  4. Proxy measures of healthcare-acquisition of MRSA such as incidence (or incidence density) of hospital-onset MRSA based on clinical culture data.
- Supplemental/advanced outcome measures for acute-care hospitals
  1. Additional measures of the burden of healthcare-associated infection (eg, incidence or incidence density of hospital-associated MRSA infections),
  2. Estimates of burden of MRSA exposure within the facility (eg, rates of overall and admission MRSA prevalence, point prevalence), and the burden of hospital-associated acquisition of MRSA (eg, incidence of hospital-onset MRSA based on clinical culture data and AST data).

In calculating these outcome measures, guidelines recommend careful consideration of how duplicate isolates from the same patient during the selected surveillance period will be handled. More specific details regarding these metrics (eg, definitions, methods of calculation) are available in the original SHEA/HICPAC position paper.^46^ In addition to calculating outcome measures locally, hospitals that report MRSA data to the CDC NHSN Multidrug Resistant Organism and *C. difficile* Infection (MDRO/CDI) Module have the option of having a number of outcome measures calculated automatically.^157^ The metrics included in this NHSN module are similar to some of those described in the SHEA-HICPAC position paper.^46^ Relative to MRSA, certain outcome measures are available to hospitals that submit only bloodstream isolate data (eg, hospital-onset MRSA bloodstream infection incidence). Additional outcomes data are available to those who submit information regarding MRSA isolates from other clinical specimens or from AST.

### External reporting: State and federal requirements

1. Federal requirements: In the United States, the Centers for Medicare & Medicaid Services (CMS) Hospital Inpatient Quality Reporting (IQR) Program requires acute-care hospitals to report hospital-wide inpatient MRSA bloodstream isolates via the CDC NHSN Multidrug-Resistant Organism and *C. difficile* Infection (MDRO/CDI) Module.^158^
2. State requirements: States may have additional reporting requirements for MRSA-related data. Contact your local or state health department for state-specific requirements.

## Section 6: Implementation strategies

Accountability is an essential principle for preventing HAIs. It provides the necessary link between science and implementation. Without clear accountability, scientifically based implementation strategies will be used in an inconsistent and fragmented way, decreasing their effectiveness in preventing HAIs. Accountability begins with the chief executive officer and other senior leaders who provide the imperative for HAI prevention, thereby making HAI prevention an organizational priority. Senior leadership is accountable for providing adequate resources needed for effective implementation of an HAI prevention program. These resources include necessary personnel (clinical and nonclinical), education, and equipment.

The information provided below is intended to assist hospitals with implementation of the essential and additional practices that they have selected for their infection prevention program. In addition to the examples provided below, please refer to the Appendix for a more detailed discussion of factors to consider during the implementation of MRSA AST and decolonization programs. Guidance for the implementation of an effective hand hygiene program is available in the Compendium document on strategies for optimizing hand hygiene.^159^

### Engage

1. Collaborate with representatives from departments and groups appropriate for the strategy being implemented (eg, hospital administration, nursing staff, medical staff, environmental services/housekeeping, facilities management, procurement, clinical laboratory, admitting and bed assignment department, case management, human resources, risk management, community and/or patient education specialists, information technology). Include opinion leaders, role models, and unit champions from these groups in planning and implementation of initiatives.
2. Consultation with a trained individual with expertise in MRSA control and prevention may be useful for program development and assessment if such a person is not available within the hospital.
3. Engage executive leadership based on clinical outcomes data, public reporting requirements, and locally determined return on investment calculations.

### Educate

1. Provide an educational program to foster desired behavior changes. Include a discussion of MRSA risk factors, routes of transmission, outcomes associated with infection, organization-specific prevention measures (and the evidence supporting their use), local MRSA epidemiology (MRSA infection rates, etc), the potential adverse effects of contact isolation, roles that HCP play in MRSA prevention, and current data regarding HCP compliance with infection prevention and control measures.
2. Target educational programs based on HCP needs (ie, healthcare practitioner, support personnel). Given the wide range of educational backgrounds and job descriptions among hospital personnel, several educational programs will be needed to provide the necessary information at the appropriate level for all relevant personnel.
3. Provide evidence that supports the use of selected strategies.
4. Education should utilize principles of adult learning (eg, use relatable case scenarios or situations) and may be accomplished in settings and formats that are determined to be the most effective by the organization, including classroom, unit-based meetings, or computer stations. Possible formats include internet-based training, newsletters, communication board postings, and other communication means. Coaching sessions, one-to-one engagement, etc, may be useful to reinforce implementation of educational materials.
5. To ensure consistent messaging to learners, consider providing standardized educational materials such as guidelines, templates, observation tools, skills training, scripting, etc., which outline minimum expectations of the organization that are relevant to the learner.

### Execute

In addition to the examples provided, please refer to the Appendix for a more detailed discussion of factors to consider during the implementation of a MRSA AST program. Guidance for the implementation of an effective hand hygiene program is available in the Compendium document on strategies for optimizing hand hygiene.^159,160^

#### MRSA monitoring program

1. A common detection strategy used by infection control programs to identify and track patients from whom MRSA has been isolated from any clinical or AST specimen includes a daily review of laboratory results to identify patients from whom MRSA has been isolated.
2. A common method of tracking MRSA is a line list:
  - The line list includes each patient’s first (and, often, subsequent) MRSA isolate, regardless of body site and includes isolates identified by clinical cultures and AST, when available.
  - Initial isolates as well as subsequent clinical infections should be classified as either hospital or community onset using prespecified definitions (see Section 2).
  - In addition, patients known to be MRSA-colonized or -infected based on testing performed at another healthcare facility should be included in the line list.
  - Additional information commonly contained in the line list includes the date of collection of specimens from which MRSA was isolated, site from which the specimen was obtained, and hospital location at the time of collection.
  - Ideally, the line list is an electronic database generated from the organization’s electronic health record, which can integrate relevant hospital data systems (eg, culture results, admissions, discharge, transfer (ADT) data, etc) to populate an electronic line list.

### Contact precautions

1. Place patients in a single or private room when available.
2. Place patients who have MRSA in cohorts when a single or private room is not available.
3. Cohort placement does not eliminate the need for compliance with hand hygiene and other infection prevention measures between patient contacts.
4. Don gown and gloves upon entry into the patient’s room and change the gown and gloves before having contact with a subsequent patient or the subsequent patient’s immediate environment.
5. HCP should have a thorough understanding of the benefits and potential adverse effects associated with the use of contact precautions.
6. Patients placed on contact precautions should continue to receive the same level and quality of care as those who are not on contact precautions.
7. Dedicate noncritical patient care items such as blood pressure cuffs, stethoscopes, etc, to a single patient when they are known to be colonized or infected with MRSA. When equipment must be shared among patients, clean and disinfect the equipment between patients.
8. Establish institutional criteria for discontinuation of contact precautions.
  - A test-based strategy may be used to determine whether a patient remains colonized with MRSA. Because a single negative surveillance test may not adequately detect the persistence of MRSA colonization, facilities may choose to require multiple negative tests prior to discontinuing contact precautions. Expert guidance is available to assist facilities in making institutional policies for discontinuation of contact precautions.^62,71^ When retesting MRSA patients to document clearance is considered, waiting at least a few months (eg, 4–6 months) since the last positive test is often advised. Some hospitals may choose to consider MRSA-colonized patients to be colonized indefinitely.

### Cleaning and disinfection

Current guidelines outline environmental and equipment disinfection and sterilization standards as follows.^61,161,162^

1. Develop written protocols for daily and terminal cleaning and disinfection of patient rooms. Protocols should address the type of equipment or surface, persons responsible for performing the tasks, frequency, disinfectant product appropriate to the device or surface, and required contact time to achieve effective disinfection.
2. Pay close attention to cleaning and disinfection of high-touch surfaces in patient care areas (eg, bed rails, carts, bedside commodes, doorknobs, and faucet handles).
3. Disinfect portable, reusable healthcare equipment after each use, at the time of patient discharge from the room in which the equipment is located, when the equipment is moved out of a room, between uses on different patients, and at the frequency recommended by the device manufacturer if specified in the instructions for use.
4. The use of supplemental disinfection methods, such as hydrogen peroxide vapor, ultraviolet light, and antimicrobial surfaces, has been shown in some non-randomized studies to have potential benefit in reducing the burden of organisms in the healthcare environment. However, these additional technologies are costly, and their clinical effectiveness for prevention of MRSA transmission has not yet been definitively proven^163–166^ Notably, these methods should be used as supplements to, but not as replacements for, routine cleaning and disinfection.

### Alert systems

1. Laboratory alerts for new MRSA-positive patients and alerts to identify MRSA-positive patients on readmission or transfer^167–169^
  - Patients with newly identified MRSA
    - The laboratory-based manual alerting system may include immediate notification of clinical and IP staff via fax, phone, pager, email, or notification in EMR or electronic surveillance system.
  - Readmission or intrafacility transfer of patients with MRSA
    - Manual or computer-based databases of patients’ MRSA status may be used to identify known MRSA-positive patients at the time of readmission and bed assignment. A designated field in the EMR may be used to indicate a patient’s MRSA-positive status.
    - The receiving unit should be notified of the patient’s MRSA-positive status prior to the patient’s arrival on the unit.
    - The alert should remain in effect until the facility’s MRSA clearance criteria have been met.
  - Interfacility transfer of patients with MRSA
    - A patient’s MRSA-positive status should be communicated to a receiving healthcare facility prior to the patient’s transfer.
    - Collaborate with nursing, discharge planning, and case management to include relevant infection control data, such as MRSA infection or colonization, on communication tools.
    - Create an infection prevention interfacility transfer tool such as the one developed by the CDC (http://www.cdc.gov/HAI/toolkits/InterfacilityTransferCommunicationForm11-2010.pdf).
    - If the patient has been transferred to another facility before susceptibility information is available, the receiving organization should be notified.
    - When receiving patients in transfer from another healthcare facility, require the transferring healthcare facility to provide MRSA status information and other relevant infection control information during the transfer hand-off communication process.

### Educating patients and their families about MRSA

1. Provide standardized information about MRSA and contact precautions. Methods of information dissemination might include patient education sheets in appropriate languages, patient education channels, websites, or video presentations. A member of the care team should assess the patient’s understanding and answer specific questions that remain.
2. Include information that addresses concerns and anticipates questions, such as general information about MRSA, the difference between colonization and infection, the hospital’s MRSA prevention program, the components of and rationale for contact precautions, and the risk of transmission to family and visitors.^74,170^
3. To alleviate MRSA-related concerns that remain after patient discharge, provide education and helpful tips about managing MRSA in the home setting.^171^
4. Determine whether educational materials will be developed by facility personnel or obtained from an external resource (eg, professional societies, public health authorities, commercial vendors).

### Active surveillance testing

Please refer to the Appendix for a more detailed discussion of the issues outlined below.

#### AST among patients

1. Select the patient population that will be included in the screening program (eg, all patients or only high-risk patients or patients on high-risk units).
2. Develop a reliable system to identify patients who meet the criteria for screening.
3. Determine how screening specimens will be ordered (eg, standardized nursing protocol, admission order set, individual patient order), who will initiate the order (eg, physician, nurse) and who will obtain the specimens (eg, unit-based nursing personnel, designated MRSA monitoring program personnel, patient).
4. Determine when screening will be performed (see Appendix).
5. Determine the anatomic sites that will be sampled.
6. Select the laboratory method that will be used to detect MRSA.
7. Determine how to manage patients while awaiting the results of screening tests
8. Assess the availability of single rooms and develop a plan and protocol for situations in which the number of single rooms is insufficient.^61,62^ When there is not a sufficient number of single rooms, the following options may be considered:
  - Prioritize patients with MRSA who are at greater risk for transmission (eg, those with draining wounds) for a single room.
  - Place MRSA colonized or infected persons in cohorts (ie, group multiple MRSA-positive patients in the same room). Ideally, MRSA patients who are cocolonized or coinfected with other MDROs should not be placed with other MRSA patients unless those patients are also cocolonized or coinfected with the same organism(s).
  - When neither placement in a single room nor cohort placement with another patient with MRSA is possible, options include keeping the patient with the existing roommate or identifying a low-risk patient with whom the MRSA-positive patient can share a room while keeping the patients physically separated (eg, keep privacy curtains drawn).^61^ Ensure that HCP have access to and use appropriate PPE for the MRSA-colonized patient and that PPE is removed and hand hygiene is performed prior to contact with the other patient or the other patient’s immediate environment.

#### AST among HCP

1. Screening of HCP is most commonly performed to mitigate and contain outbreaks. Because identified HCP carriers may serve as either a primary source of MRSA in a healthcare-associated outbreak (ie, active MRSA infection or persistent colonization with transmission to patients)^172–174^ or as a vector (secondary source) of transmission (ie, transient MRSA colonization of HCP with transmission between patients),^175–177^ it is important to be aware of these distinctions when screening programs are undertaken. Different infection prevention strategies may be more impactful if the HCP is the primary or secondary source of transmission.
2. Often, staff will be concerned about the interpretation of a positive test and whether it will identify them as the source of an outbreak. Often, the pressing goal is to contain transmission, and not to distinguish between primary and secondary sources. Conveying to staff the goal of containment over source identification can be helpful in HCP screening programs in which positive carriers are decolonized to prevent transmission to other HCP or patients regardless of the source.
3. Estimating source determination is increasingly possible due to genomic advancement. However, the goal should be to enhance practices of infection prevention to prevent spread from an ongoing common source.
4. Determine how and when to collect specimens for testing.
5. Select the laboratory method that will be used to detect MRSA.
6. Determine how to manage personnel who are identified as an ongoing primary or secondary source of MRSA transmission.

#### Decolonization therapy

1. Conduct a risk assessment to identify populations with high rates of MRSA infection that might benefit from decolonization.
2. Determine whether targeted or universal decolonization will be utilized.
  - Targeted decolonization includes AST to identify colonized individuals followed by decolonization for those with MRSA colonization.
  - Universal decolonization avoids testing and provides treatment to the entire at-risk population. This approach may provide added benefit of reducing MSSA disease in addition to MRSA disease, and it may help address concern that a single screening of limited body sites is insufficient to identify all MRSA carriers.
3. Select a decolonization regimen. (Note: Decolonization regimens typically include a combination of nasal and skin antisepsis.)
4. Consider developing standardized or protocol-based order sets to optimize compliance.
5. Standardize care processes.
6. Ensure adequate supplies of products used for decolonization (eg, chlorhexidine bottles or cloths) to reduce barriers to implementation.
7. Review chlorhexidine compatibility of patient hygiene and skin-care products and remove incompatible products that are used on the body below the neckline.
8. HCP responsible for implementing MRSA decolonization programs should receive competency-based training with return demonstration for the application of intranasal antimicrobials or antiseptics and topical CHG.^178,179^
9. Consider use of existing tool kits with protocols, education and training materials, skills assessments, and FAQs.
  - Toolkit for MRSA decolonization of non-ICU patients with indwelling devices (ABATE trial: https://www.ahrq.gov/hai/tools/abate/index.html).
  - Toolkit for implementation of universal decolonization (REDUCE MRSA trial: http://www.ahrq.gov/professionals/systems/hospital/universal_icu_decolonization/index.html).
  - Decolonization toolkit from SHIELD Orange County Project (https://www.ucihealth.org/shield).
  - Postdischarge decolonization toolkit (https://www.ucihealth.org/clearmrsa).
  - Although these tool kits were developed for specific trials, materials may be adopted for decolonization programs as outlined in the decolonization section of this document.

#### Evaluate

1. Assess compliance with infection prevention practices such as hand hygiene, gown-and-glove use, appropriate room placement, environmental cleaning and disinfection protocols, AST protocol (when applicable), and decolonization protocols (when applicable).^41,62,160,180–182^ The use of objective methods (eg, fluorescent markers and ATP detection systems) to monitor and provide feedback regarding environmental cleaning have been associated with improved thoroughness of cleaning.^183–187^ Options for evaluating environmental cleaning have been previously described.^188^
2. Review and update educational materials according to facility policies for recurring review.
  - When there are changes in process.
  - When indicated based upon feedback from healthcare staff, patient, and families.
  - When new clinical data become available.
3. Monitor MRSA outcomes.
  - For further discussion of monitoring MRSA outcomes, please refer to Section 5 where performance measures are discussed.
  - Additional resources related to MRSA outcome measures
    - CDC NHSN Multidrug-Resistant Organism and *C. difficile* Infection (MDRO/CDI) Module^45^
    - Recommendations for Metrics for Multidrug-Resistant Organisms in Healthcare Settings: SHEA/HICPAC Position Paper^46^
  - Provide HCP and hospital leadership with feedback regarding MRSA-related process and outcomes measures.
  - If decolonization is included in the MRSA prevention program, consider monitoring for the development of resistance to the agents used for decolonization (eg, mupirocin).
  - If AST among HCP is performed,
    - Assess HCP compliance with recommended screening.
    - For personnel determined to be a vector or source of MRSA outbreak, assess for compliance with the recommended prevention strategy (eg, infection control practices, decolonization therapy).
    - Assess for changes in the incidence of MRSA that are temporally associated with identification and management of colonized HCP.
    - If decolonization therapy is administered, assess the response to therapy.
      1. Consider retesting HCP who received decolonization therapy to document eradication of carriage.
      2. The optimal timing for retesting HCP who received decolonization therapy is unclear. Although no strong data support a specific approach, one relatively common approach is to retest the HCP 1–2 weeks after completion of decolonization therapy to document clearance of MRSA. Subsequent testing of the HCP to detect relapse or recurrent colonization should be considered if there is evidence of ongoing transmission despite initially successful decolonization of colonized HCP.

## Appendix

The information provided in this Appendix is intended to supplement the recommendations provided in Section 6 for the implementation of MRSA active surveillance testing and MRSA decolonization programs. Specifically, the information provided below addresses many of the complex issues that are encountered when designing and implementing these programs.

### Implementing Active Surveillance Testing (AST) Programs

#### AST among Patients

1. Select the patient population that will be included in the screening program (eg, all patients versus high-risk patients or units).
  - Use the MRSA risk assessment to determine whether all patients, patients admitted to specific high-risk units (eg, intensive care unit), or high-risk patient populations (regardless of location) will be included in the screening program. The prevalence of MRSA and the proportion of MRSA that are community-associated may influence the choice of populations to be included and the risk factors to be used in identifying patients to be screened. State legislative requirements for active surveillance, where applicable, should be considered when selecting the patient population to be screened.
  - Patient-level risk factors for MRSA colonization (eg, recent hospital or skilled nursing facility admission, chronic hemodialysis, specific upcoming surgeries, recent antimicrobial therapy) may also be used to determine inclusion in the screening program.^189–193^
  - Consider available infrastructure and hospital-specific characteristics (size, staffing for laboratory and nursing, patient population, MRSA prevalence, information technology support) when selecting the patient population(s) to be screened.
  - Consider pilot testing the program in 1 location before expanding to other locations. Select the pilot unit based on the risk or prevalence of MRSA on the unit or the presence of motivated leadership and frontline personnel.
  - Expand the program to additional units once the pilot program has been evaluated and adjusted and initial goals have been met (eg, >90% compliance with specimen acquisition).
2. Develop a reliable system to identify patients who meet the criteria for screening.
  - Identification of patients who meet criteria for MRSA screening may be more difficult when patient-level risk factors, rather than patient care unit, are used to determine inclusion in the surveillance program. Take this into consideration during the planning stages of the screening program. Hospitals with well-developed electronic medical records and other computer databases may be able to identify such patients using a computer algorithm.^189,192^
  - Consider developing and implementing a checklist to be completed at admission to assist in identifying patients to be screened for MRSA.
3. Determine how screening specimens will be ordered (eg, standardized nursing protocol, admission order set, individual patient order), who will initiate the order (eg, physician, nurse), and who will obtain the specimens (eg, unit-based nursing personnel, designated MRSA monitoring program personnel, patient).
  - These decisions will need to take into account relevant hospital policies, staffing, and infrastructure.
  - Although AST samples have historically been collected by healthcare personnel, one study that compared results of healthcare personnel-collected and patient-collected specimens demonstrated concordance rates of 82%–95% between HCP and patient-collected specimens.^194^
4. Determine when screening will be performed.
  - MRSA surveillance may be performed upon admission to the hospital, preoperatively, upon initiating dialysis, on entry to a specific unit, or upon identifying a potential outbreak.
  - Although not always included in active surveillance testing programs, additional testing of patients with initial negative surveillance test results can be done either at regular intervals (eg, weekly) or upon discharge or transfer from the hospital or unit in order detect patients who have acquired MRSA while in the hospital. This may be particularly important during MRSA outbreaks. One study demonstrated that patients identified as MRSA carriers by screening at the time of ICU discharge accounted for 27% of MRSA carriers detected by active surveillance and for 27% of the total number of MRSA colonization days in non-ICU wards for patients discharged from the ICU.^195^
  - Testing at regular intervals has the potential to detect patients who have acquired MRSA during their hospitalization earlier than testing only at discharge and thus allows implementation of contact precautions and other response activities (eg, decolonization, different empiric or prophylactic antibiotics) to prevent transmission or disease.
  - When testing is to be performed at regular intervals, consider identifying a specific day of the week when specimens will be collected. This will simplify the process and allow the microbiology laboratory to anticipate the increased volume of specimens and plan staffing and supplies accordingly.
5. Determine the anatomic sites that will be sampled.
  - The sensitivity of surveillance specimens obtained from a variety of anatomic sites has been evaluated in several settings and patient populations. Although no single site will detect all MRSA-colonized persons, most studies have found the anterior nares to be the most frequently positive site, with sensitivity ranging from 48% to 93%.^194,196–200^ Because of this and the accessibility of the site, the anterior nares have generally been considered to be the primary site for sampling in MRSA screening programs. However, collection of samples from other sites, such as skin (groin, perineum, wounds), foreign body (eg, gastrostomy or tracheostomy tube) exit sites, throat, and the perianal area, will allow identification of additional colonized patients that would not be identified by nasal specimens alone. Several recent studies have demonstrated that sampling from 1 or more additional sites, such as the throat and/or perineum, was required to increase the sensitivity of AST to >90%.^194,196,198,199^
  - The neonatal ICU has a number of unique features that should be considered when planning an AST program for that setting.^24^ For management of MRSA outbreaks in NICUs, nares samples alone may be sufficient to detect MRSA-colonized neonates,^164^ but a sampling strategy that includes collection of specimens from other sites, such as the umbilicus, may have greater sensitivity for detection of MRSA than sampling the nares alone.^125,201^
  - To simplify the specimen collection procedure and optimize resource utilization, some hospitals performing multisite sampling use a single swab to collect specimens from multiple sites (eg, nose, axillae, and groin).^166^ When using molecular-based testing methods, confirm with laboratory personnel that the test has been validated for use with all sampling sites.
6. Select the laboratory method that will be used to detect MRSA.
  - MRSA can be detected using culture-based methods or molecular diagnostic testing methods, such as polymerase chain reaction (PCR). Many factors must be considered when determining which laboratory method(s) will be used in a MRSA screening program. These factors include but are not limited to performance characteristics of the test (eg, sensitivity, specificity), batch testing, turnaround time, capabilities of the laboratory that will be providing the service (whether an in-house or reference lab), number of specimens that will be processed, and facility-specific cost-benefit calculations.
  - A detailed discussion of the various laboratory methods for MRSA detection is beyond the scope of this guideline, but some of the key features of the most common methods are discussed below.
    - Culture-based methods: Numerous microbiologic media and culture techniques have been described for use in the detection of MRSA colonization. One of the more commonly used selective media is mannitol salt agar (MSA) with or without antimicrobial (eg, oxacillin or cefoxitin) supplementation to increase specificity for methicillin-resistant organisms. The time required for detection of MRSA is ∼48 hours using most culture-based techniques. Several chromogenic agar media have been developed that allow more rapid detection of MRSA than conventional media, usually within 24 hours. Studies using established collections of isolates and clinical specimens have shown that these chromogenic media rival or outperform more conventional microbiological techniques.^202–208^ Additional enrichment steps, such as overnight incubation in trypticase soy broth, can further increase the yield of standard and chromogenic culture-based methods.^209–211^
    - Molecular testing methods: In recent years, there have been advances in molecular diagnostic testing methods, such as real-time PCR, for detection of MRSA. Earlier evaluations of these PCR assays found them to be highly sensitive (90%–100%) and specific (91.7%–98.4%) compared to standard culture-based methods.^212–215^Although more costly than culture-based techniques, one potential advantage of these molecular tests is their ability to provide a result in <2 hours from the time of specimen collection, although in actual practice the turn-around time may be longer due to batching of samples. At least 1 uncontrolled study^216^ and 3 mathematical models^217–219^ have suggested that rapid testing may allow for more effective use of contact precautions and enhanced prevention of MRSA transmission. However, a cluster randomized crossover trial of universal screening in general wards failed to identify a difference in MRSA acquisition rates with the use of rapid testing as compared with the use of a culture-based method.^52,220–222^ These data suggest that the clinical and economic benefits of rapid testing may vary among individual hospitals and settings.
7. Determine how to manage patients while awaiting the results of screening tests.^57^
  - Before implementing a screening program, a decision should be made regarding how a patient will be managed while waiting for the result of the admission MRSA screening test. There are 2 common approaches: (1) await the test result and implement contact precautions only if the screening test is positive or (2) place the patient on empiric contact precautions until a negative admission screening test result is documented.
    - It has been shown that patients colonized with MRSA often contaminate the hospital environment prior to the availability of AST results.^57^ Thus, empiric use of contact precautions could minimize the risk of MRSA transmission from unrecognized sources, and some have suggested that this approach has contributed to more effective control of MRSA.^189^ However, several logistical difficulties may be associated with this approach. Empiric use of contact precautions substantially increases the need for single rooms and the quantity of supplies needed to practice contact precautions. When only a small proportion of screened patients are colonized with MRSA and single rooms are of limited quantity, a large number of patients whose screening test results are negative will need to be moved so that their single room can be used for another patient. These room reassignments and the necessary cleaning before the vacated room can be reoccupied can impede patient flow within the hospital. In many acute-care hospitals, implementing contact precautions at the time of receipt of a positive screening test result is a reasonable initial approach. The empiric use of contact precautions for all tested patients while awaiting test results may be most feasible in hospitals where a relatively large proportion of screened patients are MRSA-positive or where a large proportion of patient rooms are single rooms and in individual hospital units, such as many ICUs, where each patient is in an individual room or bay.
    - Despite its potential logistic difficulties, empiric use of contact precautions should be considered if transmission continues despite introduction of a screening program in which contact precautions are implemented only after a positive MRSA screening test.

#### AST among HCP

1. Determine how and when to collect specimens for testing.
  - Consideration should be given to testing epidemiologically linked personnel when transmission continues despite implementation of basic control measures.
  - Data on optimal anatomic sites for screening among HCP are not readily available. There is no evidence to suggest that anatomic screening sites among personnel should be different than those sampled in patients for the purpose of detecting colonization (See Appendix). In many published reports of MRSA outbreak investigations that included AST of HCP, the nares were sampled to detect colonization. Some reports have cited sampling of other sites, either alone or in addition to the nares, including the fingertips, skin (areas of dermatitis), the perineum, and pharynx.^223^
  - The timing of collection of screening specimens may affect the results of HCP screening. Screening during or at the end of a work shift may identify transiently colonized HCP in addition to persistently colonized HCP who may be a source of ongoing transmission.^224^ Thus, collection of specimens at the beginning of a shift or after several days away from the clinical setting may optimize the specificity of testing.
  - When screening HCP for outbreak control, consider having occupational health staff inquire about or directly evaluate HCP for areas of dermatitis or any skin breakdown or wounds because these symptoms have been associated with ongoing sources of transmission, both primary and secondary. Convenience, efficiency, privacy, and comprehensive surveillance should be considered when determining whether screening occurs in a designated private location near an affected unit or work site versus in the occupational health department.
2. Select the laboratory method that will be used to detect MRSA.
  - When choosing between culture-based vs molecular based MRSA tests, be sure to evaluate whether organisms are needed for further testing, such as sequencing for clonality. Considerations regarding optimal laboratory tests for detection of MRSA carriage include the following:
    - Molecular testing (eg, pulse-field gel electrophoresis or whole-genome sequencing) to establish clonality of MRSA isolates and determine whether patient isolates and isolate(s) obtained from HCP are related has been useful in some investigations.^63,146,172,225–227^
    - Whole-genome sequencing can be integrated into outbreak investigations along with epidemiologic data to improve understanding of MRSA transmission.^228^
3. Determine how to manage personnel who are identified as an ongoing primary or secondary source of MRSA transmission.
  - Develop a facility policy to manage HCP who are either infected or colonized with an outbreak strain of MRSA in a standard fashion. Most published reports of MRSA transmission from colonized HCP have indicated that transmission was interrupted after the introduction of several simultaneous interventions.^174,223^ No controlled studies have examined the specific impact of isolated interventions on interrupting HCP to patient transmission of MRSA. Thus, there are no evidence-based recommendations for managing MRSA-colonized HCP who have been associated with ongoing MRSA transmission within a healthcare facility.
  - Consideration of the MRSA-colonized HCP’s specific job-related activities may help to determine the course of action. Interventions that may be considered include the following:
    - Evaluate the MRSA-colonized HCP’s infection prevention practices for opportunities for education and improvement. For example, in one report, an HCP with chronic sinusitis linked to a cluster of MRSA cases was identified as a carrier of the outbreak strain, and breaches in recommended infection control practices were identified.^173^
    - Ensure appropriate treatment of active MRSA infection.
    - Decolonization therapy may be considered for personnel with persistent MRSA colonization. Refer to Section 4 (Additional Strategies) and to the “Implementing Decolonization Therapy Programs” information below for further details on decolonization therapy.
  - Work restrictions: HCP work restrictions have been used as a part of outbreak management in some, but not all, reports. Work restrictions include approaches such as furlough, restriction from patient care activities, and temporary reassignment. Work restrictions have been used for some, but certainly not all, MRSA-colonized HCP who have been sources of ongoing MRSA transmission. Other approaches that have been used successfully include education and implementation of additional infection control measures. However, many outbreaks have successfully been controlled using a multimodal approach including education, treating infection, decolonization, and reinforcement of hand hygiene and have not required furlough of colonized HCPs for cessation of the outbreak.
    - Occupational health staff should make determinations for removal from work either due to active infection or carriage during a critical juncture of an uncontained outbreak.
    - If HCP are removed from work due to carriage, a protocol should be in place for reinstatement (eg, either after decolonization has been performed or after demonstrating negative screen if testing is being performed).
    - Testing for clearance of carriage should not be performed while HCP are actively on a decolonization regimen. Consider waiting 48 hours after decolonization to evaluate.

### Implementing Decolonization Therapy Programs

1. Select a decolonization regimen.
  - Decolonization regimens typically include a combination of nasal and skin antisepsis.
  - Intranasal agents
    - Mupirocin and povidone-iodine (iodophor) are the most common intranasal agents.
      1. Mupirocin is an antibiotic with a broad range of antibacterial activity, including all staphylococci. It is used clinically as an ointment or cream for topical treatment of skin infections and for nasal decolonization.^229^ Although recolonization can occur, the effect of mupirocin in eliminating MRSA carriage has been shown to be longer lasting than the effect of antiseptics for the same purpose. It is typically applied twice daily for 5 days.
      2. Iodophor (povidone-iodine) is commonly used as a skin or nasal preparation to provide antisepsis the site of use. It has a broad spectrum of activity including all staphylococci. These products are known for rapid bacterial suppression at the time of application, but suppression cannot be presumed beyond 6–8 hours.^230^ These findings suggest that the use of PI for nasal decolonization may be limited to presurgical nasal antisepsis or other applications not requiring a longer duration of effect. Studies on reapplication of povidone-iodine and duration of MRSA nasal suppression are needed.
      3. Some data comparing mupirocin and iodophor preparations for prevention of *S. aureus* infections are available.
      4. a. The Mupirocin Iodophor Swap Out Trial^231^ was a 137-hospital cluster randomized trial that evaluated the noninferiority of iodophor- chlorhexidine to mupirocin-CHG for universal ICU decolonization. Mupirocin resistance has risen over time in several geographic areas, and iodophor is an alternative nasal agent that has been predominantly used as a presurgical decolonizing agent in combination with chlorhexidine. The Mupirocin Iodophor Swap Out Trial found that mupirocin-chlorhexidine was superior, with 18% fewer *S. aureus* ICU clinical cultures and 14% fewer MRSA clinical cultures than iodophor-chlorhexidine (findings published as conference abstract). Importantly, iodophor-chlorhexidine still demonstrated benefit compared to a historical control group who did not receive universal decolonization in the REDUCE MRSA^114^ trial, which was conducted in same health system. Iodophor may be preferred in ICUs where high-level mupirocin resistance is substantial or where use of a topical nasal product that does not require a prescription is logistically important.
      5. b. A randomized trial compared a 5-day, twice-daily intranasal mupirocin (2% ointment) treatment in combination with topical chlorhexidine with 2 applications of povidone iodine (5% solution) within 2 hours of arthroplasty or spine surgery in combination with chlorhexidine on adult patients. 115 The study outcome was resultant deep-tissue S. aureus SSI within 3 months of surgery. The study group included 1,530 patients who completed the intervention. Preoperative nasal S. aureus colonization in the intent to treat group was 19% for the mupirocin group and 18% for the povidone-iodine group. The S. aureus deep SSI rate was 0.6 per 100 procedures in the mupirocin group and 0.1 per 100 procedures in the povidone-iodine group in the intent-to-treat analysis. The authors concluded that 2 applications of nasal povidone iodine within 2 hours of surgery in combination with topical CHG may be considered an alternative to 5-day, twice-daily intranasal mupirocin in combination with topical chlorhexidine and a component of a multifaceted approach to reduce SSIs.
    - Other products that have been considered for use in nasal decolonization
      1. Alcohol-based antiseptic agents developed for reducing or eliminating nasal *S. aureus* carriage have been proposed for suppression of nasal MRSA. To date, a few studies have been published evaluating a limited observation period (6–10 hours) with mixed results, but generally suggesting that alcohol may require frequent repeated application (eg, every 2–4 hours) for suppression of *S. aureus* and/or other bacteria in the nasal vestibule.
      2. a. A study evaluated alcohol-based antisepsis for nasal decolonization using 2 nonblinded randomized trials.^232^ In one trial, a single-dose application versus triple-dose application of commercial alcohol-based nasal antisepsis was used with MRSA colonized patients. Specimens were collected 3 times over an 8-hour interval for MRSA detection. The single-dose applications of an alcohol-based sanitizer did not significantly reduce nasal MRSA. The one-time triple-dose application only transiently reduced MRSA, with no significant reduction by 6 hours after application of the product. In a separate trial, a triple-dose application was spread over 8 hours. Specimens were collected 2 hours after the final dose with no reduction in nasal MRSA, which again suggested that even short term (2–4 hours) suppression effect requires multiple reapplication.
      3. b. A randomized controlled trial studied the reduction of nasal *S. aureus* carriage in healthcare professionals who were *S. aureus* carriers.^233^ A commercially available alcohol-based nasal antiseptic product was applied at 4-hour intervals over the duration of a typical work day (8–10 hours). Antiseptic treatment reduced *S aureus* colony-forming units (CFUs) from baseline by 99% (median) and 82% (mean) (*P* < .001). The antiseptic or placebo was reapplied at 4-hour intervals. Total bacterial CFUs were reduced by 91% (median) and 71% (mean) (*P* < .001) over the 10-hour period. This study does elucidate the transient timeframe of bacterial suppression in the study population of healthcare professionals. However, the time frames of suppression and the applicability for patient decolonization were not verified. These factors require further study and careful consideration regarding clinical situations in which it may be of some use.
  - Topical agents
    - The most commonly available skin antiseptic in the United States is chlorhexidine gluconate (CHG), a divalent cation containing 2 biguanides. CHG provides antiseptic activity by causing disruption of microbial cell membranes and precipitation of cell contents. Depending on its concentration, it is bacteriostatic (inhibits bacterial growth) or bactericidal (kills bacteria). It is highly effective against gram-positive organisms and is often used as a skin antiseptic to reduce the risk of *S. aureus* (MSSA and MRSA) infections.
    - Trials of products available outside of the United States are available for other skin antiseptic products. These products have not been used in the large decolonization trials referred to in this document but are worth mentioning as they are similar to CHG and are being studied in other countries.
      1. Olanexidine gluconate (OLG) is a monovalent cation possessing only 1 biguanide. A microbiologic study by Nakaminami et al^234^ showed that the fast-acting bactericidal activity of OLG against qacA/B-positive MRSA is greater than that of CHG and is equivalent to the bactericidal action of povidone iodine. A review of studies of the pharmacological and clinical effects suggests that the clinical usefulness of OLG in prevention of surgical site infections and device-related infections requires additional study.^235^
      2. Octenidine dihydrochloride is a cationic biguanide with a broad antibacterial spectrum toward gram-positive bacteria including MRSA. Octenidine is not percutaneously absorbed and partly remains on the location of the application, providing persistence at that site. It has been used in both MRSA skin and nasal antisepsis protocols. Intranasal application of octenidine together with universal antiseptic bathing was shown to reduce the prevalence and acquisition of MRSA colonization in extended-care facilities in Singapore.^236^ Also in Singapore, a study involving MRSA prevention in a high-prevalence acute-care dermatology ward, patients admitted during intervention period who received additional topical intranasal octenidine were 63% less likely to acquire MRSA than those receiving universal daily octenidine bathing alone during baseline period.^237^ Denkel et al^238^ performed a randomized controlled trial that compared CHG and octenidine with routine care on the prevention of central-line–associated bloodstream infections in 72 ICUs in Germany. Compared to routine bathing, neither showed significant preventive effect on CLABSI rates, although the authors stated that the study risked being underpowered; CLABSI rates in routine care group were lower than initially assumed.^238^
    - Other products
      1. Other products that have been suggested for skin antisepsis in decolonization regimens include tea-tree oil, sodium hypochlorite (bleach), triclosan, and others.^239^ More studies to illuminate the evidence on use of these products in healthcare protocols are needed.
2. Standardize care processes.
  - Determine the method of chlorhexidine application. A variety of chlorhexidine products are available for skin antisepsis:
    - Single-use bottles of aqueous chlorhexidine that can be applied as 4% rinse-off CHG in the shower or be added to a basin of water and diluted 1:1 to make 2% no-rinse CHG for bed bathing.
    - Manufacturers of 2% no-rinse chlorhexidine-impregnated cloths for bed bathing.
    - It should be noted that the use of undiluted no-rinse 4% aqueous chlorhexidine solution for skin cleansing has been associated with a relatively high rate of reversible adverse skin effects (eg, skin fissures, itching, and burning of the skin),^240^ for this reason, 2% CHG should be used for no-rinse applications, as this concentration has been widely used in several large-scale clinical trials with minimal side effects.
    - Skin concentrations of chlorhexidine are inversely associated with bacterial microbial density on the skin, suggesting a benefit for ensuring that application achieves effective microbicidal skin concentrations.^241^
  - Issues to consider when selecting chlorhexidine products may include available supporting clinical data, cost, ease of use, and consistency of application.
  - Follow the manufacturer’s recommendations when using a chlorhexidine-containing product. These recommendations include avoidance of direct contact with nervous tissue, including direct contact with the eyes and middle ear (eg, in patients with perforated tympanic membranes).
  - Chlorhexidine is used widely in children aged <2 months old.^136^ HCP must carefully weigh the potential benefit in preventing MRSA related-outcomes in children aged <2 months old and preterm infants and the risks of CHG, recognizing that term and preterm infants may have different risks.^115,242^ For chlorhexidine gluconate-based topical antiseptic products, the Food and Drug Administration recommends “Use with care in premature infants or infants under 2 months old; these products may cause irritation or chemical burns.” Concerns in children aged <2 months old include skin irritation and systemic absorption following topical exposure, events that may be more likely in preterm infants in the first month of life.^136^
  - Provide physical barriers to prevent chlorhexidine solution from depositing onto linens to minimize staining when linens contact bleach oxidizers during commercial laundering.
  - If the decolonization regimen will include intranasal application of mupirocin, determine how mupirocin will be provided (eg, in single-dose or multidose tubes).
